# Supplementary material for: Prospective cohort study to identify prevalence, risk factors and outcomes of infection associated kidney disease in a regional hospital in Malawi
Source: BMJ Open. 2022 Nov 28;12(11):e065649. doi: 10.1136/bmjopen-2022-065649 (PMC9710333; doi:10.1136/bmjopen-2022-065649)
Supplement: Supplementary data [file bmjopen-2022-065649supp001.pdf]

Title: Prospective cohort study to identify prevalence, risk factors and outcomes of infection associated kidney disease in a regional hospital in Malawi.

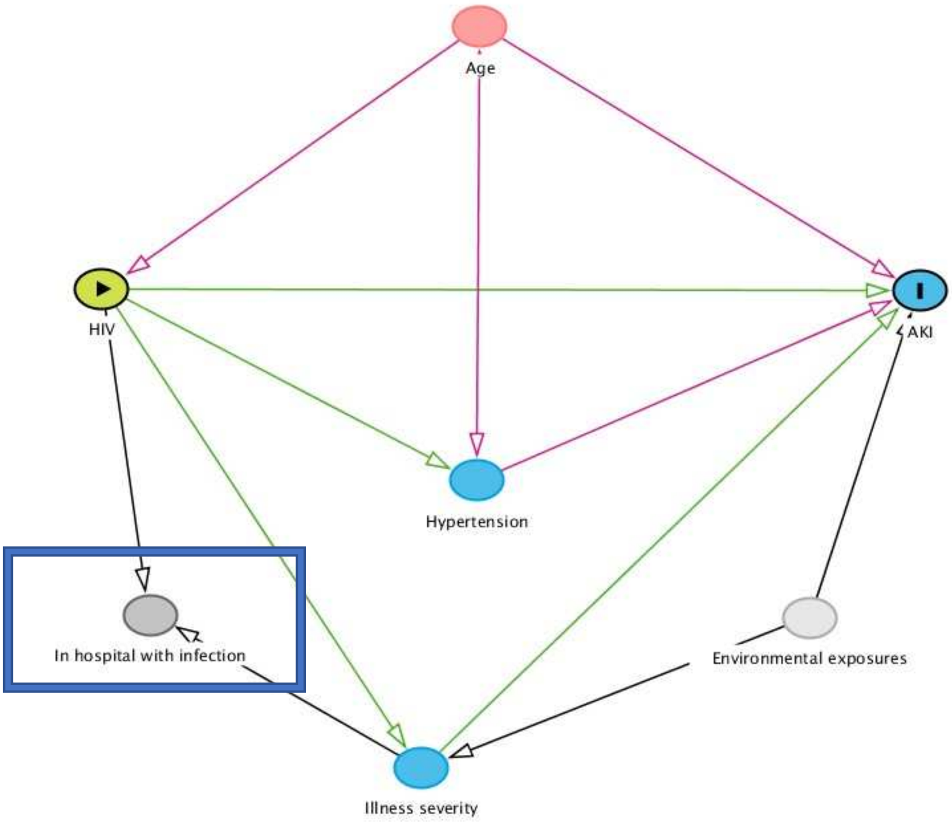

Supplementary figure 1: directed acyclic graph*.
Demonstrates the hypothesized causal relationship for examining the total effect of HIV exposure on acute kidney injury and requires adjustment for age. Note existence of collider variable *in hospital with infection*, conditioned on due to study design, by recruiting patients hospitalised with infection.
*created using DAGitty[1].

Carey LI, *et al. BMJ Open* 2022; 12:e065649. doi: 10.1136/bmjopen-2022-065649

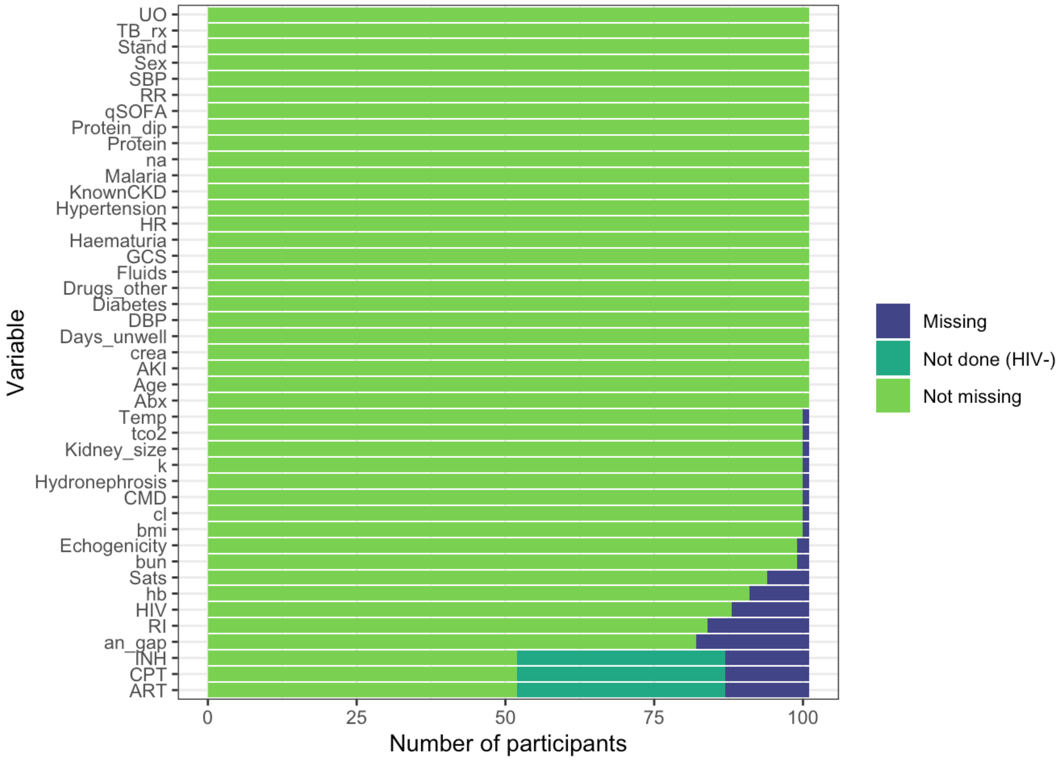

Supplementary figure 2: **missing data by variable.** HIV related variables were not available for adults without HIV, recorded as "Not done (HIV-)". Urine = able to pass urine), TB_rx = history of TB treatment, SBP = systolic blood pressure, RR = respiratory rate, HR = heart rate, drugs_other = received any over the counter or traditional medications, DBP = diastolic blood pressure, Abx = received antibiotics prior, stand = able to stand, K = serum potassium, CMD = corticomedullary differentiation, bicarb = bicarbonate, BUN = blood urea nitrogen, Sats = capillary oxygen saturations, Hb = haemoglobin, RI = renal resistive index, INH = isoniazid, CPT = cotrimoxazole preventive therapy, ART = antiretroviral therapy.

Carey LI, *et al. BMJ Open* 2022; 12:e065649. doi: 10.1136/bmjopen-2022-065649

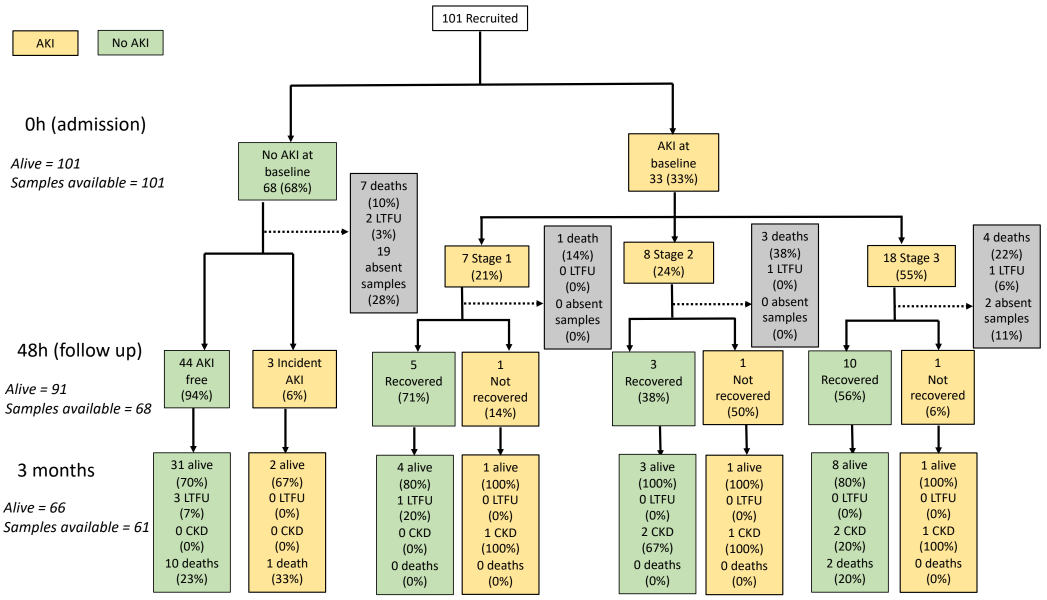

Supplementary figure 3: study flow diagram. Adults admitted to Zomba District Hospital with community acquired infection were recruited (n=101). AKI was defined using the KDIGO criteria based on a 1.5 fold increase in admission creatinine from estimated baseline, stratified by stages 1-3. AKI recovery was assessed at 48h (decrease of 0.3 mg/dL between baseline and 48 hours or a normal creatinine (< 1.3 mg/dL) at 3 months). New (incident) AKI was assessed at 48 hours and diagnosed according to KDIGO using estimated baseline creatinine. CKD was assessed at 3 months and defined as an estimated glomerular filtration rate < 60 mL/min/1.73/m^2 (composite of both CKD-EPI and MDRD equation).
LTFU = lost to follow up.

Carey LI, *et al. BMJ Open* 2022; 12:e065649. doi: 10.1136/bmjopen-2022-065649

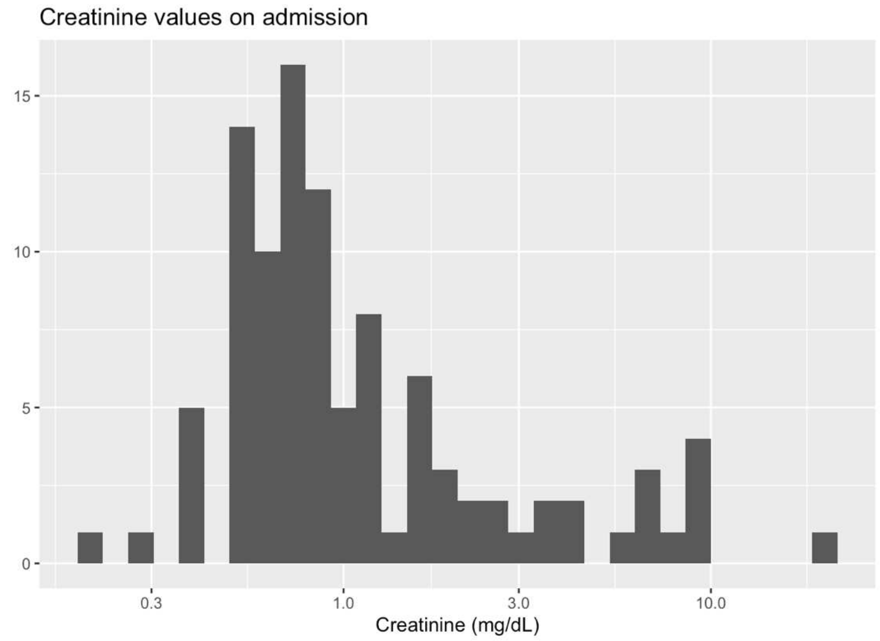

Supplementary figure 4: histogram of baseline creatinine values in mg/dL (log scale).

a

HIV -ve	qSOFA score		
**Age (years)**	**1**	**2**	**3**
**20**	0.09	0.30	1.00
**40**	0.02	0.09	0.30
**60**	0.09	0.30	1.00
**80**	0.82	2.85	10.11

b

HIV +ve	qSOFA score		
**Age (years)**	**1**	**2**	**3**
**20**	0.45	1.50	5.25
**40**	0.12	0.43	1.50
**60**	0.45	1.50	5.25
**80**	4.26	13.29	49.00

Supplementary figure 5
a AKI odds for HIV negative participants with diastolic blood pressure held constant at 80 mm Hg

Carey LI, *et al. BMJ Open* 2022; 12:e065649. doi: 10.1136/bmjopen-2022-065649

b AKI odds for people living with HIV with diastolic blood pressure held constant at 80 mm Hg. Colour coding yellow to orange depicts increasing odds.

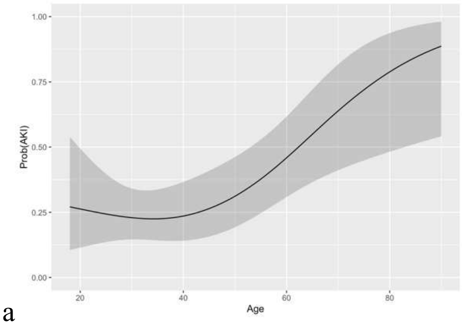

a

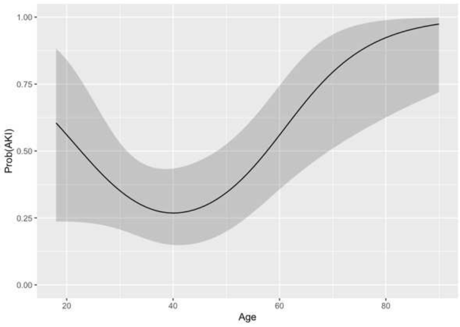

b

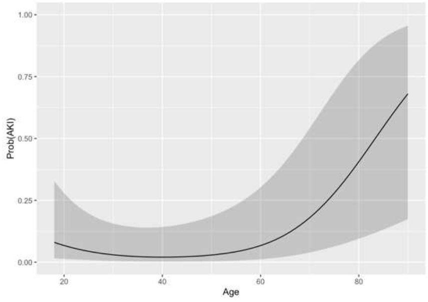

c

Supplementary figure 6
a unadjusted effect of age on probability of AKI. Age is expressed as a spline term with 3 knots.
b adjusted effect of age on probability of AKI. Diastolic blood pressure = 75 mm Hg, qSOFA score = 2, HIV status = positive.
c Adjusted effect of age on probability of AKI. Diastolic blood pressure = 75 mm Hg, qSOFA score = 1, HIV status = negative.

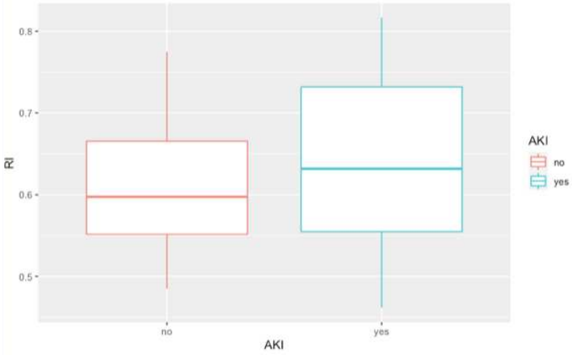

Supplementary figure 7: boxplot of RI distributions by AKI status
RI = renal resistive index.

Supplementary table 1: ultrasound, laboratory and derived indices.

Variable		Value
Ultrasound	Kidney Size (cm)	10 [9-10]
	Hydronephrosis* n/N (%)	16/100 (16%)
	Loss of corticomedullary differentiation* n/N (%)	44/100 (44%)
	Increased echogenicity† n/N (%)	41/99 (41%)
	Renal Resistive Index	0.62 [0.55-0.68]
Urine	Able to pass urine n/N (%)	97/101 (96%)
	Proteinuria on dipstick n/N (%)	29/101 (29%)
	Proteinuria (mg/dL)	0.0 [0.0-138.5]
	Microscopic Haematuria n/N (%)	54/101 (53%)
Observations	Systolic blood pressure (mm Hg)	117 [103-133]
	Diastolic blood pressure (mm Hg)	75 [64-84]
	Heart rate (beats/min)	101 [84-123]
	Temperature (°C)	37 [37-38]
	Oxygen saturation (%)	98 [96-99]
	Respiratory rate (breaths/min)	24 [22-28]
	Glasgow coma score < 15 n/N (%)	27/101 (27%)
Illness severity	Unable to stand unaided n/N (%)	27/101 (27%)
	Length of time unwell for (days)	7.0 [3.0-21.0]
	Receiving intravenous fluid n/N (%)	63/101 (62%)
	qSOFA score	2.0 [2.0-2.0]
Laboratory parameters	Haemoglobin (mmol/L)	11.9 [8.8-13.9]
	Blood Urea Nitrogen (mmol/L)	15 [8-30]
	Chloride (mmol/L)	104 [99-108]
	Sodium (mmol/L)	136 [131-140]
	Potassium (mmol/L)	3.6 [3.3-4.5]
	Bicarbonate (mmol/L)	23 [19-26]
	Creatinine (mmol/L)	0.80 [0.60-1.60]
	Anion Gap (mmol/L)	16 [15-18]

Values are median [interquartile range].
* Hydronephrosis/corticomedullary differentiation missing for 1 participant n=100
† Echogenicity missing n=2

Carey LI, *et al. BMJ Open* 2022; 12:e065649. doi: 10.1136/bmjopen-2022-065649

Supplementary table 2: demographic univariable associations with AKI status.

Variable	No AKI	AKI	*p* value
Age† (years)	35.75 [28.62-46.06]	46.46 [35.44-65.43]	0.01
Male sex* n (%)	32 (47)	15 (45)	1.00
Body mass index (kg m^{-2})	22.06 [19.38-23.63]	20.96 [18.87-23.23]	0.19
Creatinine mg/dL	0.7 (0.5-0.8)	2.7 (1.6-6.3)	<0.01**
HIV infected* n (%)	33 (55)	20 (71)	0.29
Receiving antiretroviral therapy* n (%)	26 (79)	16 (84)	0.39
Receiving co-trimoxazole preventative therapy* n (%)	25 (76)	15 (79)	0.52
Receiving isoniazid preventative therapy* n (%)	6 (18)	5 (26)	0.33
Received TB treatment* n (%)	6 (9)	5 (15)	0.33
Prior Malaria* n (%)	12 (18)	9 (27)	0.30
Diagnosis of Diabetes* n (%)	0 (0)	1 (3)	0.33
Diagnosis of Hypertension* n (%)	2 (3)	5 (15)	0.04
Antibiotic use prior* n (%)	51 (75)	29 (88)	0.19
Over the counter/traditional meds* n (%)	2 (3)	2 (6)	0.60
Vomiting* n (%)	14 (21)	11 (33)	0.22
Diarrhoea* n (%)	10 (15)	6 (18)	0.77
Cough* n (%)	26 (38)	17 (52)	0.28

Values are median [interquartile range].
* Fisher's exact test † Wilcoxon rank sum

Carey LI, *et al. BMJ Open* 2022; 12:e065649. doi: 10.1136/bmjopen-2022-065649

Supplementary table 3: ultrasound and urine related univariable associations with AKI status.

**Variable**	**No AKI**	**AKI**	***p* value**
Kidney Size† (cm), median (IQR)	9.8 (8.96-10.49)	9.63 (8.67-10.35)	0.65
Hydronephrosis*, n (%)	9 (13%)	7 (21%)	0.39
Loss of corticomedullary differentiation*, n (%)	28 (42%)	16 (48%)	0.53
Increased echogenicity*, n (%)	25 (38%)	16 (48%)	0.29
Renal Resistive Index†, median (IQR)	0.6 (0.55-0.66)	0.63 (0.56-0.73)	0.19
Able to pass urine*, n (%)	67 (99%)	30 (91%)	0.10
Proteinuria on dipstick*, n (%)	11 (16%)	18 (55%)	<0.01
Proteinuria† (mg/dL), median (IQR)	0 (0-26.4)	182.1 (65.4-241.7)	<0.01
Microscopic Haematuria*, n (%)	27 (40%)	27 (82%)	<0.01

Values are median [interquartile range].
* Fisher's exact test † Wilcoxon rank sum

Carey LI, *et al. BMJ Open* 2022; 12:e065649. doi: 10.1136/bmjopen-2022-065649

Supplementary table 4: observation and laboratory derived univariable associations according to AKI status.

Variable	No AKI	AKI	*p* value
Temperature† (°C)	37.2 [36.85-37.8]	37 [36.8-37.8]	0.58
Heart rate† (beats/min)	99.5 [83.75-124]	102 [92-123]	0.51
Respiratory rate† (breaths/min)	23.5 [20-26.25]	26 [23-29]	0.08
Systolic blood pressure† (mm Hg)	115.5 [103.75-128.25]	118 [101-141]	0.28
Diastolic blood pressure† (mm Hg)	74 [64-83.25]	77 [63-87]	0.35
Oxygen saturation† (%)	98 [96-99]	97 [95.5-98]	0.07
Glasgow coma score < 15*, n (%)	17 (25)	10 (30)	0.63
Unable to stand unaided*, n (%)	15 (22)	12 (36)	0.15
Length of time unwell† (days)	9 [3-30.44]	5 [3-14]	0.18
Receiving intravenous fluid*, n (%)	38 (56)	25 (76)	0.08
qSOFA score†	2 [2-2]	2 [2-2]	0.27
Haemoglobin† (mmol/L)	11.9 [9.2-13.75]	11.55 [8.1-14.6]	0.88
Blood Urea Nitrogen† (mmol/L)	10 [7-17.5]	40.5 [22.75-82.25]	<0.01
Chloride† (mmol/L)	102.5 [98-108]	105.5 [101.75-109.75]	0.09
Sodium† (mmol/L)	136 [130.75-140]	136 [131-140]	0.99
Potassium† (mmol/L)	3.6 [3.3-4.2]	3.6 [3.17-5.18]	0.80
Bicarbonate† (mmol/L)	24 [22-26]	16 [14-23]	<0.01
Creatinine† (mg/dL)	0.7 [0.5-0.8]	2.7 [1.6-6.3]	<0.01
Anion Gap† (mmol/L)	16.5 [15-18]	16.5 [15-20]	0.24
eGFR† (ml/min/1.73/m^2) (MDRD)	124.04 [92.68-159.04]	22.95 [11.21-40.24]	<0.01
Creatinine clearance† (mL/min) (Cockroft and Gault)	118.25 [93.71-143.71]	24.88 [14.48-33.66]	<0.01
Chronic kidney disease at 3 months*, n (%)	5 (12)	7 (37)	0.06

Values are median [interquartile range].
* Fisher's exact test † Wilcoxon rank sum
MDRD = 4-parameter Modification of Diet in Renal Disease equation

Carey LI, *et al. BMJ Open* 2022; 12:e065649. doi: 10.1136/bmjopen-2022-065649

Supplementary Table 5: Effect of age on AKI when full age spectrum equation is used to back calculate creatinine, using assumed eGFR 100.

Odds ratios and 95% confidence intervals for AKI		
**Age in years**	**Unadjusted**	**Adjusted for age and HIV status**
20	1.17 (0.38-5.63)	2.23 (1.28-38.11)
30	0.91 (0.57-1.68)	0.91 (0.71-2.34)
40 (ref)	1.00	1.00
50	1.92 (1.45-3.07)	2.03 (1.49-4.43)
60	5.43 (3.22-20.11)	8.72 (4.21-171.31)
70	17.46 (7.20-189.53)	64.33 (18.65-28395.00)
80	56.58 (15.97-2103.49)	529.11 (96.75-5708002.00)

Supplementary Table 6: Effect of age on AKI and AKD according to each definition on mortality*.

	**MDRD back calculation assumed eGFR 100 mL/min**		***p* value**
	**No AKI**	**AKI**	
Death by 3 months, n (%)	18/68 (26%)	10/33 (30%)	0.70
	**Full age spectrum equation assumed eGFR 100 mL/min**		***p* value**
	**No AKI**	**AKI**	
Death by 3 months, n (%)	17/65 (26%)	11/36 (30%)	0.60
	**Lowest creatinine as baseline**		***p* value**
	**No AKI**	**AKI**	
Death by 3 months, n (%)	28/84 (33%)	0/17 (0%)	<0.01
	**Acute kidney disease**		***p* value**
	**No AKD**	**AKD**	
Death by 3 months, n (%)	11/38 (29%)	17/63 (27%)	0.70

**p* values obtained by Chi squared test of significance.
AKD = acute kidney disease, AKI = acute kidney injury

Carey LI, *et al. BMJ Open* 2022; 12:e065649. doi: 10.1136/bmjopen-2022-065649

Reference

1. Textor J, van der Zander B, Gilthorpe MS, Liśkiewicz M, Ellison GTH. Robust causal inference using directed acyclic graphs: the R package 'dagitty.' *Int J Epidemiol*. Published online January 15, 2017:dyw341. doi:10.1093/ije/dyw341
