# Supplementary material for: Prospective cohort study to identify prevalence, risk factors and outcomes of infection associated kidney disease in a regional hospital in Malawi
Source: BMJ Open. 2022 Nov 28;12(11):e065649. doi: 10.1136/bmjopen-2022-065649 (PMC9710333; doi:10.1136/bmjopen-2022-065649)
Supplement: Author's manuscript [file bmjopen-2022-065649.draft_revisions.pdf]

BMJ Open is committed to open peer review. As part of this commitment we make the peer review history of every article we publish publicly available.

When an article is published we post the peer reviewers' comments and the authors' responses online. We also post the versions of the paper that were used during peer review. These are the versions that the peer review comments apply to.

The versions of the paper that follow are the versions that were submitted during the peer review process. They are not the versions of record or the final published versions. They should not be cited or distributed as the published version of this manuscript.

BMJ Open is an open access journal and the full, final, typeset and author-corrected version of record of the manuscript is available on our site with no access controls, subscription charges or pay-per-view fees (http://bmjopen.bmj.com).

If you have any questions on BMJ Open's open peer review process please email info.bmjopen@bmj.com

BMJ Open

Risk factors for acute kidney injury and outcomes among sepsis admissions in Malawi.

Journal:	*BMJ Open*
Manuscript ID	bmjopen-2022-065649
Article Type:	Original research
Date Submitted by the Author:	14-Jun-2022

Complete List of Authors:	Carey, Laura; Liverpool School of Tropical Medicine, Clinical Sciences; Malawi-Liverpool-Wellcome Trust Clinical Research Programme,<br>Kaimba, Sylvester; Malawi-Liverpool-Wellcome Trust Clinical Research Programme<br>Nyirenda, Saulos; Zomba Central Hospital, Medicine<br>Chetcuti, Karen; Kamuzu University of Health Sciences<br>Joekes, Elizabeth; Liverpool School of Tropical Medicine, Clinical Sciences<br>Henrion, Marc; Malawi-Liverpool-Wellcome Trust Clinical Research Programme, ; Liverpool School of Tropical Medicine,<br>Rylance, Jamie; Liverpool School of Tropical Medicine, Clinical Sciences; Malawi-Liverpool-Wellcome Trust Clinical Research Programme,

Keywords:	Epidemiology < TROPICAL MEDICINE, Acute renal failure < NEPHROLOGY, Adult nephrology < NEPHROLOGY, Nephrology < INTERNAL MEDICINE

SCHOLARONE™
Manuscripts

I, the Submitting Author has the right to grant and does grant on behalf of all authors of the Work (as defined in the below author licence), an exclusive licence and/or a non-exclusive licence for contributions from authors who are: i) UK Crown employees; ii) where BMJ has agreed a CC-BY licence shall apply, and/or iii) in accordance with the terms applicable for US Federal Government officers or employees acting as part of their official duties; on a worldwide, perpetual, irrevocable, royalty-free basis to BMJ Publishing Group Ltd ("BMJ") its licensees and where the relevant Journal is co-owned by BMJ to the co-owners of the Journal, to publish the Work in this journal and any other BMJ products and to exploit all rights, as set out in our licence.

The Submitting Author accepts and understands that any supply made under these terms is made by BMJ to the Submitting Author unless you are acting as an employee on behalf of your employer or a postgraduate student of an affiliated institution which is paying any applicable article publishing charge ("APC") for Open Access articles. Where the Submitting Author wishes to make the Work available on an Open Access basis (and intends to pay the relevant APC), the terms of reuse of such Open Access shall be governed by a Creative Commons licence – details of these licences and which Creative Commons licence will apply to this Work are set out in our licence referred to above.

Other than as permitted in any relevant BMJ Author's Self Archiving Policies, I confirm this Work has not been accepted for publication elsewhere, is not being considered for publication elsewhere and does not duplicate material already published. I confirm all authors consent to publication of this Work and authorise the granting of this licence.

For peer review only

AUTHORS

Laura Carey*[1,2] Sylvester Kaimba[1], Saulos Nyirenda[3], Karen Chetcuti[4,5], Elizabeth Joekes[2,5], Marc Y. R. Henrion[1,2], Jamie Rylance[1,2]

1 Malawi Liverpool Wellcome Clinical Research Programme, Blantyre, PO Box 30096, Malawi

2 Department of Clinical Sciences, Liverpool School of Tropical Medicine, Liverpool, UK, L3 5QA

3 Zomba Central Hospital, Malawi Ministry of Health, Zomba, Malawi

4 Kamuzu University of Health Sciences, Private Bag 360, Chichiri, Blantyre 3

5 Worldwide Radiology, Liverpool, UK

***Corresponding author**

Dr. Laura Carey

Liverpool School of Tropical Medicine

Pembroke Place

Liverpool

L3 5QA

E: lcarey@mlw.mw

T: +447745279825

TITLE

Risk factors for acute kidney injury and outcomes among sepsis admissions in Malawi.

Word count: 3716

For peer review only

ABSTRACT

Objectives: Acute kidney injury (AKI) is a common and severe complication of sepsis, but data on impact in sub-Saharan Africa (SSA) are lacking. We determined AKI prevalence, risk factors and outcomes in adults with sepsis in Malawi.

Design: A prospective cohort study of sepsis patients, from Feb-Jun 2021, collecting demographic, clinical, laboratory and renal ultrasonography data.

Setting: Adults admitted to a combined secondary/tertiary hospital in Southern Region, Malawi.

Primary and secondary outcome measures: The primary outcomes were AKI prevalence and mortality by Cox proportional hazard model. AKI was defined according to Kidney Disease Improving Global Outcomes (KDIGO) guidelines. Secondary outcomes were risk factors for AKI identified by logistic regression.

Results: We recruited 101 patients recruited with sepsis. Median age was 38 years [IQR 29-48]), 88 had known HIV status of which 53 (60%) were HIV-infected, and of these 42 (79%) were receiving antiretroviral therapy. AKI was present in 33/101, of which 18/33 (55%) were severe (KDIGO stage 3). At 3 months 35/101 (35%) participants had died, while 12/61 (20%) survivors had chronic kidney disease. AKI was independently associated with qSOFA score (OR 3.10, 95% CI 1.09-8.84), older age (age 60 versus 40: OR 4.53, 95% CI 2.24-14.66), and HIV positivity (OR 4.87, 95% CI 1.38-17.13). After adjustment for age and qSOFA, living with HIV was independently associated with death (HR 3.91, 95% CI 1.06-14.48).

Conclusions: AKI is common among sepsis admissions in Malawi and associated with sepsis severity, with a large proportion progressing to chronic kidney disease at 3 months. Sepsis management protocols should identify high risk patients for focussed intensive prevention measures given the limited options for renal replacement therapy.

Word count: 271/300

For peer review only - http://bmjopen.bmj.com/site/about/guidelines.xhtml

STRENGTHS AND LIMITATIONS

- We provide data on prevalence, risk factors, and consequences of acute kidney injury (AKI), which are scarce in sub-Saharan Africa (SSA).
- We make use of the expanding availability of point of care ultrasound in Malawi and assess its role in in the AKI assessment.
- This is the first AKI study in Malawi reporting outcomes up to 3 months enabling ascertainment of chronic kidney disease (CKD) status.
- eGFR equations and methods for estimating baseline creatinine have not been validated in Malawian populations.
- The study was not powered to detect small effect sizes (OR <3.79).

INTRODUCTION

Globally, 1.7 million people die from acute kidney injury (AKI) every year, 80% of deaths are in low and middle income countries[1]. Sepsis, a common cause of AKI in sub-Saharan Africa (SSA), is a life threatening organ dysfunction triggered by a dysregulated host response to infection[2]. AKI in SSA is severe; 10-70% of presentations are stage 3, requiring renal replacement therapy, with an inpatient mortality of 44%[3,4]. The incidence of AKI among sepsis admissions in Malawi is unknown. No data exist for outcomes beyond hospital discharge, meaning that incidence of newly acquired chronic kidney disease (CKD) following an AKI episode are also unknown.

The International Society of Nephrology (ISN) initiative aims that 'no one should die of untreated AKI in low resource regions by 2025'[5]. In SSA, access to renal function testing is limited, contributing to delayed AKI diagnosis and potential progression to kidney failure. In the region, patients are typically younger than those from high-income countries, and the majority will have no prior diagnosis or creatinine ascertainment. Available conventional estimators of baseline renal function underestimate AKI incidence e.g. using the Modification of Diet in Renal Disease

For peer review only - http://bmjopen.bmj.com/site/about/guidelines.xhtml

(MDRD) equation to back-calculate creatinine assuming an eGFR of 75 ml/L/1.73m^2 underestimates AKI incidence by >50%[6].

Bedside investigations such as ultrasound may be used to identify those at increased risk of AKI, through assessment of renal morphology, or by doppler estimation of the Renal Resistive Index (RI) which directly correlates with intra-renal arterial resistance. The potential and expanding availability of ultrasound in Low- and Middle-Income Countries (LMICs) led us to assess RI and other sonographic renal parameters in the diagnosis and risk-stratification of AKI in sepsis.

We aimed to estimate the prevalence of AKI and subsequent CKD in non-hospital-acquired sepsis in Malawian adults. Secondarily, we evaluated risk factors for AKI and outcomes of mortality from sepsis episodes.

METHODS

Zomba Central Hospital (ZCH) is a regional hospital in Southern Malawi serving both urban and rural populations with sixty adult medical inpatient beds. Local HIV prevalence is estimated at 16.8% among 15-49 year olds[7].

At ZCH, we performed a prospective observational study recruiting adults (≥18 years) with suspected infection (reporting in the last week any of: fever; rigors; night sweats; treatment with antibiotics), and a medical decision to admit to hospital. All patients in the admissions unit with suspected infection were approached for screening, Monday-Friday, 0700-1700.

Exclusion criteria were any of: people lacking capacity to consent, with no proxy consent available; pregnancy; non-severe uncomplicated localised infection (such as cellulitis); detainees; trauma; admission to surgical or obstetric/gynaecological ward; previously recruitment to the study. Responsibility for care and treatment decisions remained with the usual clinical team. Participants were followed-up as inpatients at 48 hours, and at 3 months in the community.

For peer review only - http://bmjopen.bmj.com/site/about/guidelines.xhtml

Sampling and Laboratory methods

Patients provided blood and urine at baseline, 48 hours, and 3 months. Creatinine, blood urea nitrogen (BUN), glucose, ionized calcium, sodium, potassium, chloride, bicarbonate, were assessed by iSTAT CHEM8 cartridges (Abbott Point of Care Inc., Princeton, NJ). The handheld iSTAT system measures creatinine using an enzymatic assay traceable to the U.S NIST Standard Reference material SRM909 with a reportable range of 0.20-20.0 mg/dL. Standard of care for measurement of serum creatinine in ZCH is using a sarcosine oxidase (enzymatic) method on a Mindray BS360-E machine. Often this test is unavailable due to reagent stock outs which are supplied by the Government.

Point of care HIV testing was done for those with unknown status or no recent negative test, and CD4 cell count quantification. Urine was tested by dipstick for blood and protein, and positive samples further quantified using a laboratory analyser (Mindray BS360).

Ultrasound

Point of care renal ultrasound assessed kidney size, corticomedullary differentiation, echogenicity, and presence of any hydronephrosis. Assessment was made by a clinical officer who underwent dedicated training, including direct supervision by a consultant radiologist before study commencement. RI was measured at each pole of each kidney (upper, middle, lower) and an average taken across poles and between kidneys giving one RI value per participant. A portable Mindray DP-50 machine with 5MHz convex probe was used throughout.

AKI assessment

AKI was defined according to KDIGO as a 1.5 fold increase in creatinine from estimated baseline[8]. Urine output was not assessed as it is not universal clinical practice in Malawi. AKI severity as fold increase over estimated baseline was defined as: stage 1 (1.5-1.9x); stage 2 (2.0-2.9x); stage 3 ($\geq$3.0x).

For peer review only - http://bmjopen.bmj.com/site/about/guidelines.xhtml

AKI associated with infection, particularly in LMICs, affects patients unlikely to have a documented baseline creatinine, necessitating estimation through back-calculation[6]. Given the young age distribution of the population with relatively few comorbidities, baseline creatinine was estimated using a modified Modification of Diet in Renal Disease (MDRD) equation, assuming a glomerular filtration rate (GFR) of 100 mL/min/1.73m^2[6]. MDRD was modified to exclude the race coefficient as eGFR equations using ethnicity adjustment factors overestimate GFR, leading to underdiagnosis of CKD and negative bias in people of Black ethnicity[9–11]. A sensitivity analysis was done to assess the impact of changing the assumed GFR from 100 mL/min/1.73m^2 to 75 mL/min/1.73m^2.

Creatinine was measured on admission, at 48-hours and at 3-month follow up. AKI was defined as community acquired if meeting KDIGO criteria on admission. New (incident) AKI was assessed at 48h and diagnosed according to KDIGO using estimated baseline creatinine. AKI recovery was assessed at 48h and defined as recovered if either partial (creatinine within 50% of estimated baseline) or complete (creatinine returned to within 15% of estimated baseline).

Sepsis severity

Sepsis severity was measured by quick sepsis related organ failure assessment score (qSOFA) which uses three criteria: one point for low blood pressure (systolic blood pressure ≤ 100 mm Hg), high respiratory rate (≥22 breaths per minute), or altered mental state (Glasgow coma scale <15).

Outcomes

Mortality was defined as death by 48 hours or 3 months. CKD was defined as an eGFR of <60 mL/min/1.73m^2 at 3-months calculated using the 4-parameter Modification of Diet in Renal Disease (MDRD) study equation, for a non-IDMS-traceable creatinine method, without race adjustment[12–14]. MDRD estimates GFR adjusted for body surface area[12].

Statistical Analysis

For peer review only - http://bmjopen.bmj.com/site/about/guidelines.xhtml

Statistical analyses were performed in R (v4.0.2)[13,15]. Summary statistics were calculated for the cohort which was then divided into AKI and no-AKI groups, described using median and interquartile range (IQR) for continuous variables and proportions for categorical variables. Two sample t-tests or non-parametric tests, depending on data distribution were used to compare variables between groups, and Fisher's test for proportions.

Possible risk factors for AKI were considered for logistic regression analysis. Key variables were selected through a hypothesised causal structure of the relationship between sepsis and AKI (supplementary figure 1). Supplementary figure 1a demonstrates a model with CKD and qSOFA as covariates of interest. This model was not used in the final regression model as no participants reported pre-existing CKD and we did not have access to previous serum creatinine results. Supplementary figures 1b and 1c represent the same model with a single covariate at a time. Supplementary figure 1c was used as the final model with qSOFA as the covariate of interest, and age, HIV, and hypertension as secondary covariates. Logistic regression outputs were reported as odds ratio (OR) and 95% confidence intervals.

Age was included in the adjusted model as a spline term with 3 knots. The effect of age on probability of AKI was modelled using predicted probabilities from the adjusted model (when qSOFA score = 2, HIV status = positive, and diastolic blood pressure = 75 mm Hg). Odds ratios for different ages were generated by comparing odds to the reference age, chosen here to be 40 years[16]. Bootstrap sampling and the percentile method with 1,000 replicates were then used to construct 95% confidence intervals for the odds ratio.

Kaplan-Meier survival analysis was conducted using Cox proportional hazards stratified by AKI status and adjusted for age and qSOFA score. HRs for AKI were not reported as the proportional hazards assumption was not met.

Sample Size

For peer review only - http://bmjopen.bmj.com/site/about/guidelines.xhtml

A power calculation for specific risk factors was not performed before initiation of this exploratory study. However, a post-hoc power calculation was performed in G Power v3.1, given the achieved sample size (88 participants with recorded HIV and AKI status), using HIV as the risk factor[17]. Given the observed prevalence of AKI in HIV negative participants at 23% (8/35) with HIV prevalence at 60% (53/88), for a two tailed test, with 80% power and 0.05 alpha, the study was powered to detect effect sizes with an OR at least as large as 3.79.

Missing data

Missing data, <18% across all variables (supplementary figure 2), were imputed using multiple imputation with chained equations (using the 'mice' package in R) generating 5 datasets[18]. Sensitivity analyses were performed by conducting a complete case analysis.

Patient and public involvement

At the end of the study period, participants, patients, and staff at ZCH were invited to a kidney awareness day, where key results were disseminated using simple leaflets, presentations, and a group discussion.

RESULTS

Between 3rd February 2021 and 19th July 2021, 101 eligible patients with sepsis were recruited. The study flow chart is summarised in supplementary figure 3. Table 1 summarises the baseline characteristics of the participants. There were 47/101 (47%) male participants. The median age was 38 (interquartile range [IQR] 29-48). Median follow up time was 92 days (IQR 71-95). Median number of days unwell prior to hospital presentation was 7 (IQR 3-21).

Table 1: characteristics of participants.

Variable		Value
Demographics	Age (years)	38 [29-48]
	Male sex	47/101 (47)

For peer review only - http://bmjopen.bmj.com/site/about/guidelines.xhtml

	BMI	22 [19-23]
HIV/TB/Malaria	HIV infected*	53/88 (60%)
	Receiving antiretroviral therapy†	42/53 (79%)
	Receiving co-trimoxazole preventative therapy†	40/53 (75%)
	Receiving isoniazid preventative therapy†	11/53 (21%)
	History of receiving TB treatment	11/101 (11%)
	Prior Malaria	21/101 (21%)
Comorbidities	Pre-existing diagnosis of diabetes mellitus	1/101 (1%)
	Pre-existing diagnosis of hypertension	7/101 (7%)
Drugs	Antibiotic use prior	80/101 (79%)
	Traditional/OTC medications	4/101 (4%)
Symptoms	Vomiting	25/101 (25%)
	Diarrhoea	16/101 (15%)
	Cough	43/101 (43%)

Values are median [interquartile range].
* HIV status missing for 16 participants
†ART/co-trimoxazole/isoniazid status missing for 1 participant
OTC over the counter medications, *ART* antiretroviral therapy

For those who HIV status was known (88/101), 53/88 (60%), of participants were people living with HIV (PLHIV). Among PLHIV, 42/53 (79%) were receiving ART, 40/53 (76%) co-trimoxazole preventive therapy, and 11/53 (21%) isoniazid preventive therapy. Among all participants recruited, 11/101 (11%) had a history of receiving treatment for tuberculosis and 21/101 (21%) for malaria within the past month. The majority, 80/101 (79%), reported antibiotic use prior to presentation and 4/101 (4%) reported using over the counter medications or traditional medicines. No participants had a known prior diagnosis of CKD, 1/101 (1%) reported a history of diabetes, and 7/101 (7%) a history of hypertension.

Ultrasound, laboratory, and derived indices are summarised in supplementary table 1. Participants were classified according to AKI status. Using assumed GFR of 75 and 100 ml/min/1.73m^2 to estimate baseline creatinine, 28 (28%) and 33 (33%) respectively met the definition for AKI. Outcomes are presented in table 2. The

For peer review only - http://bmjopen.bmj.com/site/about/guidelines.xhtml

prevalence of CKD among survivors with creatinine values available at 3 months was 12/61 (20%).

Table 2: GFR, AKI, outcome, and CKD variables

Variable		Value
GFR	eGFR (mL/min/1.73/m^2) MDRD	93 [41-141]
	Creatinine clearance (mL/min) (Cockroft and Gault)	94 [35-129]
AKI	Acute Kidney Injury at presentation (assumed GFR 100 mL/min/1.73m^2)	33/101 (33%)
	Acute Kidney Injury at presentation (assumed GFR 75 mL/min/1.73m^2)	28/101 (28%)
	Stage 3 AKI	18/33 (55%)
	Stage 2 AKI	8/33 (24%)
	Stage 1 AKI	7/33 (21%)
	Incident AKI at 48h§	3/80 (4%)
Outcomes	Alive at 48 hours‡	91/94 (97%)
	Alive at 3 months‡	66/94 (70%)
CKD	Chronic kidney disease (eGFR < 60 ml/min/1.73m^2) at 3 months¶	12/61 (20%)

Values are median [interquartile range].
§ 48h creatinine missing n = 21
‡ Lost to follow up n = 7
¶ 3 month creatinine missing in survivors n = 5

Demographic univariable associations with AKI status are summarised in supplementary table 2, ultrasound and urine related univariable associations are presented in supplementary table 3 and observation and laboratory univariable associations in supplementary table 4. We reported a higher frequency of renal abnormalities identified on point-of-care ultrasound in participants with AKI compared to those without AKI. Kidneys were smaller in size, with a higher proportion of hydronephrosis, loss of corticomedullary differentiation and increased echogenicity; albeit not reaching the threshold for statistical significance.

For peer review only - http://bmjopen.bmj.com/site/about/guidelines.xhtml

Table 2 shows the prevalence of CKD at 3 months out of 61 available samples: in the AKI group it was 7/19 (37%) compared to 5/42 (12%) in the no-AKI group ($p = 0.06$).

Multivariable associations with AKI status (based on estimated baseline creatinine of GFR 100 mL/min/1.73m^2) identified by logistic regression are presented as unadjusted and adjusted ORs and 95% confidence intervals (CIs) in table 3.

Table 3: **Logistic regression analysis of risk factors associated with AKI**. Parameter estimates expressed as unadjusted and adjusted ORs and 95% confidence intervals.

**Risk factor**	**Unadjusted OR (95% CI) single variable model [p value]**	**Adjusted* OR (95% CI) Multivariable model [p value]**
Age in years		
20	1.07 (0.25-4.26)	1.98 (0.54-25.56)
30	0.91 (0.52-1.61)	1.14 (0.67-3.28)
40 (ref)	1	1
50	**1.63 (1.14-2.21)**	**1.70 (1.13-2.53)**
60	**3.45 (1.60-7.20)**	**4.53 (2.24-14.66)**
70	**8.07 (2.24-30.72)**	**14.17 (4.38-136.57)**
80	**18.93 (3.14-137.70)**	**44.65 (8.85-1530.58)**
Sex - Male	0.94 (0.40-2.18) [0.88]	-
HT diagnosis	**5.89 (1.05-32.90) [0.04]**	-
HIV positive	1.99 (0.77-5.15) [0.15]	**4.87 (1.38-17.13) [0.01]**
BMI	0.96 (0.86-1.07) [0.49]	-
Diastolic BP	(1.0-1.04) [0.10]	**1.03 (1.00-1.06) [0.03]**
HCO3	**0.78 (0.70-0.87) [<0.01]**	-
SpO2	0.90 (0.77-1.05) [0.18]	-
qSOFA	1.52 (0.72-3.2) [0.27]	**3.10 (1.09-8.84) [0.03]**
Microscopic haematuria	**6.83 (2.46-18.99) [<0.01]**	-
Proteinuria	**6.22 (2.40-16.13) [<0.01]**	-
RI	**24.38 (1.17-507.41) [0.04]**	-
Kidney size (cm)	0.84 (0.58-1.25) [0.40]	-
Loss of CMD	1.27 (0.54-2.95) [0.58]	-
Increased echogenicity	1.56 (0.66-3.66) [0.31]	-

For peer review only - http://bmjopen.bmj.com/site/about/guidelines.xhtml

*Multivariable model based on hypothesised reality (supplementary figure 1c) with qSOFA (measure of sepsis severity) as covariate of interest, adjusted for age, HIV status, and diastolic blood pressure. As secondary covariates, effect estimates for age, HIV status and diastolic should not be interpreted as direct effect estimates.

Sepsis severity, indicated by qSOFA score, is an independent risk factor for AKI, after adjusting for age, HIV status, and diastolic blood pressure (OR 2.94 95% CI 1.03-8.41). Increasing age, HIV status and diastolic blood pressure also appear to be associated with AKI, however require interpretation as adjustment covariates rather than direct effect estimates[19]. Supplementary figure 4 presents these findings as a forest plot of risk factors for AKI with adjusted ORs and 95% confidence intervals. Supplementary figure 5 shows the odds of AKI for different ages, qSOFA scores and HIV status, with systolic blood pressure held constant at 80 mm Hg.

The effect of age on probability of AKI is shown in supplementary figure 6. Boxplot distributions of RI values according to AKI status are in supplementary figure 7. Findings were confirmed on complete case analysis (supplementary table 5). Time-to-event analysis, adjusted for age and qSOFA score and stratified by AKI status, demonstrated an increased hazard of death for HIV positive participants, (HR 3.91, 95% CI 1.06-14.48) (Figure 1 Kaplan-Meier curves).

DISCUSSION

Adults presenting with sepsis in Zomba, Malawi are young, and predominantly living with HIV. Compared with high income settings, where AKI frequently represents renal injury acquired in hospital, our patients had evidence of kidney injury at the point of admission. Of those with sepsis and AKI, the majority was severe (stage 3), with a greater proportion of incident CKD at 3 months compared to those without AKI (37% compared to 12%). Sepsis severity (measured by qSOFA) was an independent risk factor for AKI and after age adjustment, HIV positivity was independently associated with death at 3 months. Most deaths occurred post discharge (30% versus 3% within 48 hours), indicating long term mortality associated with sepsis and AKI episodes in Malawi.

For peer review only - http://bmjopen.bmj.com/site/about/guidelines.xhtml

To our knowledge, this is the first AKI study in Malawi to obtain creatinine values at 3 months post discharge enabling ascertainment of CKD status following AKI admissions. We do not know the true prevalence of CKD at presentation, given there were no historical serum creatinine results and patients did not report known CKD, precluding CKD as a covariate in multivariable models. However, CKD prevalence of this cohort on presentation is likely to be low given the age demographic. As a small exploratory study, our study was not powered to detect moderate or small associations. GFR estimates are less accurate in the non-steady state such as AKI. For example, estimated GFR overestimates measured GFR when serum creatinine is rising. Urine output was not used to define AKI which may have led to underestimation and imprecision of AKI staging. Ascertainment of CKD status relied on serum creatinine without quantification of albuminuria(18-23). It was not possible to ascertain causes of death. We present combinations of risk factors and their effect on odds of AKI in supplementary figure 5, but we propose that future work involve the development and validation of a formal risk prediction model.

Patients presenting with sepsis in Malawi do not have documented baseline creatinine values. Back-calculation is therefore done using GFR equations. This becomes problematic, because the optimal equation to accurately estimate GFR in Malawi (and across SSA) is uncertain. In studies from SSA, coefficients for African-American ethnicity consistently over-estimate GFR[22]. Similarly, applying creatinine based eGFR formulae in HIV without adjusting for BMI tends to overestimate GFR and therefore underestimate CKD burden[10]. We estimated baseline creatinine using an assumed eGFR of 100 ml/min/1.73m^2, which resulted in a 5% difference in AKI incidence compared to an assumed eGFR of 75 ml/min/1.73m^2. Population mean eGFRs in urban Malawian adults of a similar age suggest it is reasonable to assume an eGFR>90 ml/min/1.73m^2[20]. How our incident CKD estimates at 3 months compare to the community population prevalence of CKD in Malawi is not known. A cross-sectional prevalence survey of urban and rural Malawi suggest eGFR<60ml/min/1.73m^2 of 1.4% (95% CI 1.1-1.7) and eGFR<90ml/min/1.73m^2 of

For peer review only - http://bmjopen.bmj.com/site/about/guidelines.xhtml

20.6% (19.5-21.7)[20]. According to a recent systematic review, the estimated pooled prevalence for CKD across 13 countries in SSA is 14%[21].

We reported a higher frequency of renal abnormalities identified on point-of-care ultrasound in participants with AKI compared to those without AKI. However, the study was likely underpowered to detect differences in corticomedullary differentiation, size, and echogenicity between groups (supplementary table 3). Higher RI values were associated with AKI in the unadjusted logistic regression model (table 3), but not in the univariate, non-parametric Wilcoxon rank-sum test, suggesting that the association may be driven by the larger RI values in the AKI group (supplementary table 3 and supplementary figure 7). RI values can be technically challenging to obtain and are not commonly practiced in Malawi, and we note the wide confidence interval around our OR estimate (OR 24.38 with 95% CI 1.17-507.41). While the data suggest, they do not comprehensively support their use in an AKI assessment and further investigations, with larger datasets, are needed to interrogate this potential association.

Our study used point of care creatinine testing providing immediate bedside results, however, renal function testing is frequently significantly delayed in Malawi. This may be due to malfunctioning laboratory equipment, interrupted electricity, and supply of consumables. Given limited availability of renal replacement therapy, timely diagnosis of AKI is essential for limitation and progression of renal injury. To prevent deaths from untreated AKI in low resource regions by 2025, diagnosis of AKI needs to be made as early as possible in the hospital admission[1]. Adults surviving severe AKI should be counselled on how to prevent future episodes: such as avoiding nephrotoxic medications which are commonly used in Malawi (for example non-steroidal anti-inflammatories). Given the need to back calculate when ascertaining AKI status, the ideal eGFR equation for estimating baseline creatinine in Malawians needs to be determined. The African Research on Kidney Disease (ARK) study is currently comparing direct GFR measurement using iohexol excretion with existing eGFR equations among sub-Saharan Africans, including a cohort from Malawi[23]. Interventions to reduce the impact of AKI should focus limited resources on high-risk

For peer review only - http://bmjopen.bmj.com/site/about/guidelines.xhtml

individuals, including those with HIV. This should include outpatient follow up to prevent CKD progression and end-stage kidney failure given the scarcity of renal replacement therapy.

FUNDING

LC was funded by an institutional translational partnership award [grant 219633/Z/19/Z] from the Wellcome Trust to Malawi-Liverpool-Wellcome Trust Clinical Research Programme (MLW). JR is funded by a Wellcome Career Development Fellowship [grant 206545/Z/17/Z] and by the National Institute for Health Research (NIHR) (17/63/42) using UK aid from the UK Government to support global health research. The views expressed are those of the authors and not necessarily those of the National Health Service, NIHR or the Department of Health and Social Care. For the purpose of open access, the author has applied a CC BY public copyright licence to any Author Accepted Manuscript version arising from this submission.

COMPETING INTERESTS

The authors declare no competing interests.

AUTHOR CONTRIBUTIONS

LC and JR conceived and designed the study. SN was involved in the conceptualization and project administration. EJ and KC were involved in conceptualisation and point of care ultrasound and KC delivered the ultrasound training. SK collected the data and performed the ultrasounds. LIC analysed the data and wrote the manuscript. MYRH advised on statistics. All authors read, commented on, and approved the final manuscript.

DATA AVAILABILITY

For peer review only - http://bmjopen.bmj.com/site/about/guidelines.xhtml

For peer review only

Data and code available at https://github.com/careyla/PARIS

ETHICAL APPROVAL

Participants gave written informed consent under ethical approvals from the College of Medicine Research Ethics Committee, University of Malawi (P.03/19/2625) and the Liverpool School of Tropical Medicine Ethics Committee (18-062). Study information including purposes, benefits and risk was provided to all participants in both English and Chichewa. All methods and reporting follow STROBE (Strengthening and Reporting of Observational Studies in Epidemiology) guidelines[25].

REFERENCES

1 Mehta RL, Cerdá J, Burdmann EA, *et al.* International Society of Nephrology's 0by25 initiative for acute kidney injury (zero preventable deaths by 2025): a human rights case for nephrology. *Lancet Lond Engl* 2015;**385**:2616–43. doi:10.1016/S0140-6736(15)60126-X

2 Singer M, Deutschman CS, Seymour CW, *et al.* The Third International Consensus Definitions for Sepsis and Septic Shock (Sepsis-3). *JAMA* 2016;**315**:801–10. doi:10.1001/jama.2016.0287

3 Olowu WA, Niang A, Osafo C, *et al.* Outcomes of acute kidney injury in children and adults in sub-Saharan Africa: a systematic review. *Lancet Glob Health* 2016;**4**:e242-250. doi:10.1016/S2214-109X(15)00322-8

4 Evans RDR, Hemmilä U, Craik A, *et al.* Incidence, aetiology and outcome of community-acquired acute kidney injury in medical admissions in Malawi. *BMC Nephrol* 2017;**18**:21. doi:10.1186/s12882-017-0446-4

5 Mehta RL, Burdmann EA, Cerdá J, *et al.* Recognition and management of acute kidney injury in the International Society of Nephrology 0by25 Global Snapshot: a multinational cross-sectional study. *Lancet Lond Engl* 2016;**387**:2017–25. doi:10.1016/S0140-6736(16)30240-9

For peer review only - http://bmjopen.bmj.com/site/about/guidelines.xhtml

6 Cooper DJ, Plewes K, Grigg MJ, *et al.* An Evaluation of Commonly Used Surrogate Baseline Creatinine Values to Classify AKI During Acute Infection. *Kidney Int Rep* 2021;**6**:645–56. doi:10.1016/j.ekir.2020.12.020

7 Gupta-Wright A, Corbett EL, Oosterhout JJ van, *et al.* Rapid urine-based screening for tuberculosis in HIV-positive patients admitted to hospital in Africa (STAMP): a pragmatic, multicentre, parallel-group, double-blind, randomised controlled trial. *The Lancet* 2018;**392**:292–301. doi:10.1016/S0140-6736(18)31267-4

8 Khwaja A. KDIGO clinical practice guidelines for acute kidney injury. *Nephron Clin Pract* 2012;**120**:c179-184. doi:10.1159/000339789

9 Gama RM, Clery A, Griffiths K, *et al.* Estimated glomerular filtration rate equations in people of self-reported black ethnicity in the United Kingdom: Inappropriate adjustment for ethnicity may lead to reduced access to care. *PloS One* 2021;**16**:e0255869. doi:10.1371/journal.pone.0255869

10 Glaser N, Deckert A, Phiri S, *et al.* Comparison of Various Equations for Estimating GFR in Malawi: How to Determine Renal Function in Resource Limited Settings? *PLOS ONE* 2015;**10**:e0130453. doi:10.1371/journal.pone.0130453

11 Eastwood JB, Kerry SM, Plange-Rhule J, *et al.* Assessment of GFR by four methods in adults in Ashanti, Ghana: the need for an eGFR equation for lean African populations. *Nephrol Dial Transplant* 2010;**25**:2178–87. doi:10.1093/ndt/gfp765

12 Levey AS, Coresh J, Greene T, *et al.* Using standardized serum creatinine values in the modification of diet in renal disease study equation for estimating glomerular filtration rate. *Ann Intern Med* 2006;**145**:247–54. doi:10.7326/0003-4819-145-4-200608150-00004

13 Pattaro C. *nephro: Utilities for Nephrology*. 2017. https://CRAN.R-project.org/package=nephro (accessed 6 Mar 2022).

14 Diao JA, Wu GJ, Taylor HA, *et al.* Clinical Implications of Removing Race From Estimates of Kidney Function. *JAMA* 2021;**325**:184–6. doi:10.1001/jama.2020.22124

15 R: The R Project for Statistical Computing. https://www.r-project.org/ (accessed 24 May 2022).

16 Shepherd BE, Rebeiro PF, Caribbean, Central and South America network for HIV epidemiology. Brief Report: Assessing and Interpreting the Association Between Continuous Covariates and Outcomes in Observational Studies of HIV Using Splines. *J Acquir Immune Defic Syndr 1999* 2017;**74**:e60–3. doi:10.1097/QAI.0000000000001221

For peer review only - http://bmjopen.bmj.com/site/about/guidelines.xhtml

17 Faul F, Erdfelder E, Buchner A, *et al.* Statistical power analyses using G*Power 3.1: Tests for correlation and regression analyses. *Behav Res Methods* 2009;**41**:1149–60. doi:10.3758/BRM.41.4.1149

18 Buuren S van, Groothuis-Oudshoorn K, Vink G, *et al. mice: Multivariate Imputation by Chained Equations*. 2021. https://CRAN.R-project.org/package=mice (accessed 6 Mar 2022).

19 Westreich D, Greenland S. The table 2 fallacy: presenting and interpreting confounder and modifier coefficients. *Am J Epidemiol* 2013;**177**:292–8. doi:10.1093/aje/kws412

20 Nakanga WP, Prynn JE, Banda L, *et al.* Prevalence of impaired renal function among rural and urban populations: findings of a cross-sectional study in Malawi. *Wellcome Open Res* 2019;**4**:92. doi:10.12688/wellcomeopenres.15255.1

21 Stanifer JW, Jing B, Tolan S, *et al.* The epidemiology of chronic kidney disease in sub-Saharan Africa: a systematic review and meta-analysis. *Lancet Glob Health* 2014;**2**:e174-181. doi:10.1016/S2214-109X(14)70002-6

22 Fabian J, George JA, Etheredge HR, *et al.* Methods and reporting of kidney function: a systematic review of studies from sub-Saharan Africa. *Clin Kidney J* 2019;**12**:778–87. doi:10.1093/ckj/sfz089

23 Kalyesubula R, Fabian J, Nakanga W, *et al.* How to estimate glomerular filtration rate in sub-Saharan Africa: design and methods of the African Research into Kidney Diseases (ARK) study. *BMC Nephrol* 2020;**21**:20. doi:10.1186/s12882-020-1688-0

24 Namazzi R, Batte A, Opoka RO, *et al.* Acute kidney injury, persistent kidney disease, and post-discharge morbidity and mortality in severe malaria in children: A prospective cohort study. *eClinicalMedicine* 2022;**44**. doi:10.1016/j.eclinm.2022.101292

25 Field N, Cohen T, Struelens MJ, *et al.* Strengthening the Reporting of Molecular Epidemiology for Infectious Diseases (STROME-ID): an extension of the STROBE statement. *Lancet Infect Dis* 2014;**14**:341–52. doi:10.1016/S1473-3099(13)70324-4

For peer review only - http://bmjopen.bmj.com/site/about/guidelines.xhtml

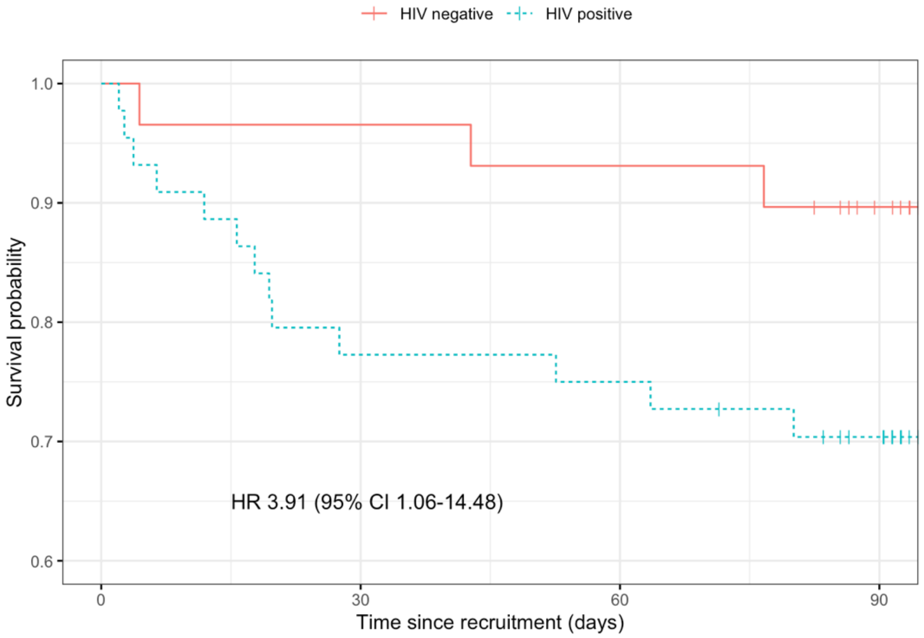

a

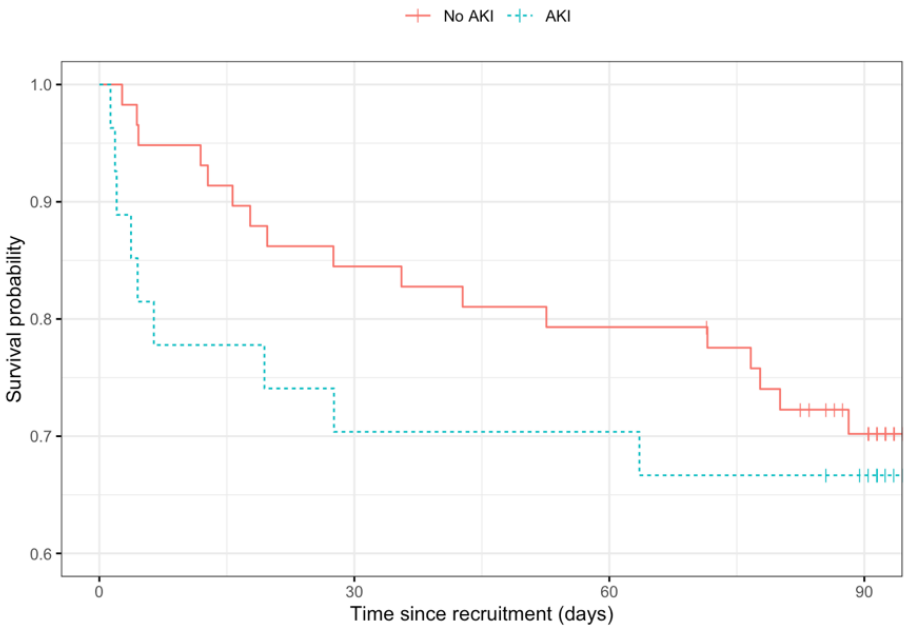

b

Figure 1: **Kaplan-Meier estimate of survival function following sepsis admission.**

a HR for death according to HIV status from Cox proportional hazards model adjusted for age and qSOFA score and stratified by AKI status with 95% confidence intervals.

b HR for death according to AKI status not reported when stratified by AKI status (proportional hazard assumption does not hold). HR = hazard ratio.

Title: Risk factors for acute kidney injury and outcomes among sepsis admissions in Malawi.

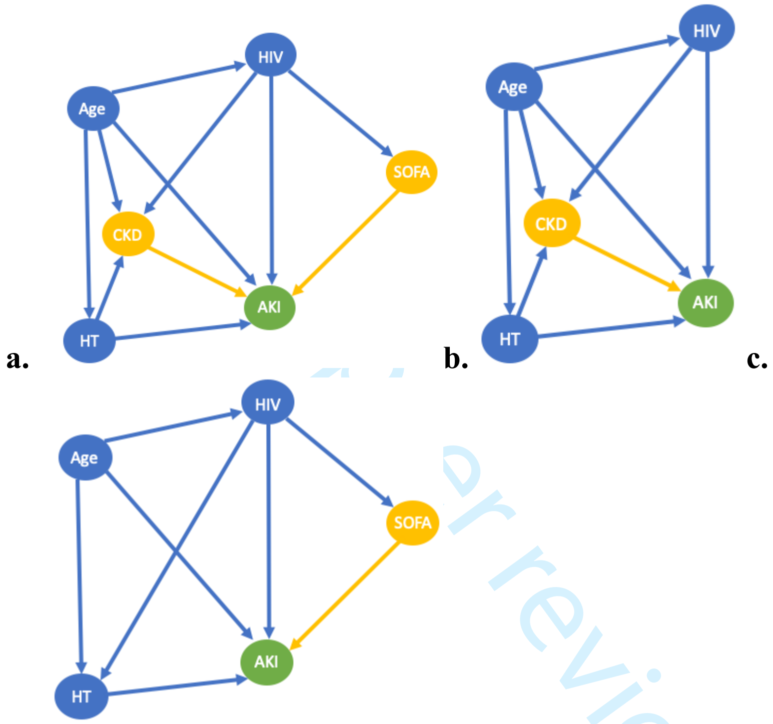

Supplementary figure 1: hypothesized causal structure for AKI risk factors in sepsis.

a. Model includes CKD and qSOFA as covariates. Sepsis severity is indicated by qSOFA score. HIV, age and hypertension are secondary covariates.
b. Model includes CKD as covariate of interest, with HIV, age and hypertension are secondary covariates.
c. Model includes qSOFA as covariate of interest, with HIV, age and hypertension are secondary covariates.

CKD = chronic kidney disease, qSOFA = quick sequential organ failure assessment, HT = hypertension

For peer review only

For peer review only - http://bmjopen.bmj.com/site/about/guidelines.xhtml

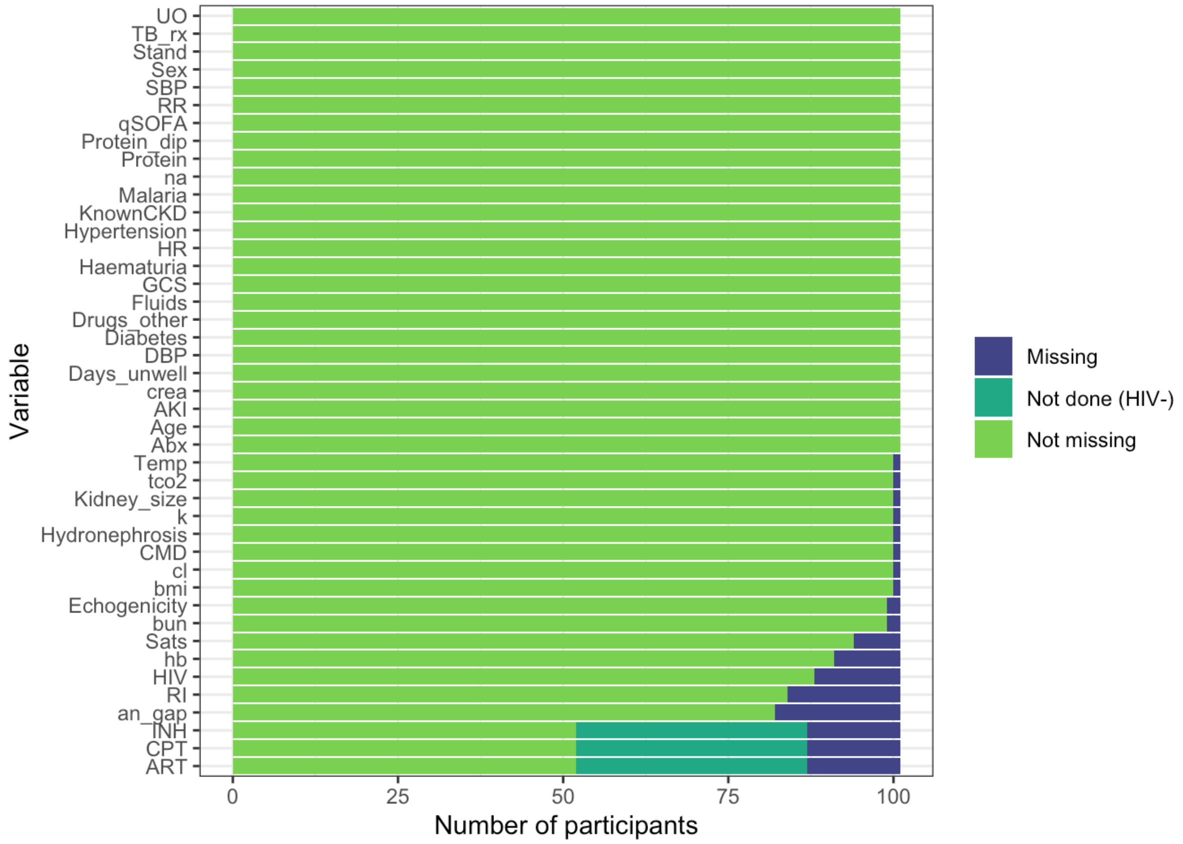

Supplementary figure 2: **missing data by variable.** HIV related variables were not available for adults without HIV, recorded as "Not done (HIV-)". Urine = able to pass urine), TB_rx = history of TB treatment, SBP = systolic blood pressure, RR = respiratory rate, HR = heart rate, drugs_other = received any over the counter or traditional medications, DBP = diastolic blood pressure, Abx = received antibiotics prior, stand = able to stand, K = serum potassium, CMD = corticomedullary differentiation, bicarb = bicarbonate, BUN = blood urea nitrogen, Sats = capillary oxygen saturations, Hb = haemoglobin, RI = renal resistive index, INH = isoniazid, CPT = cotrimoxazole preventive therapy, ART = antiretroviral therapy.

review only

For peer review only - http://bmjopen.bmj.com/site/about/guidelines.xhtml

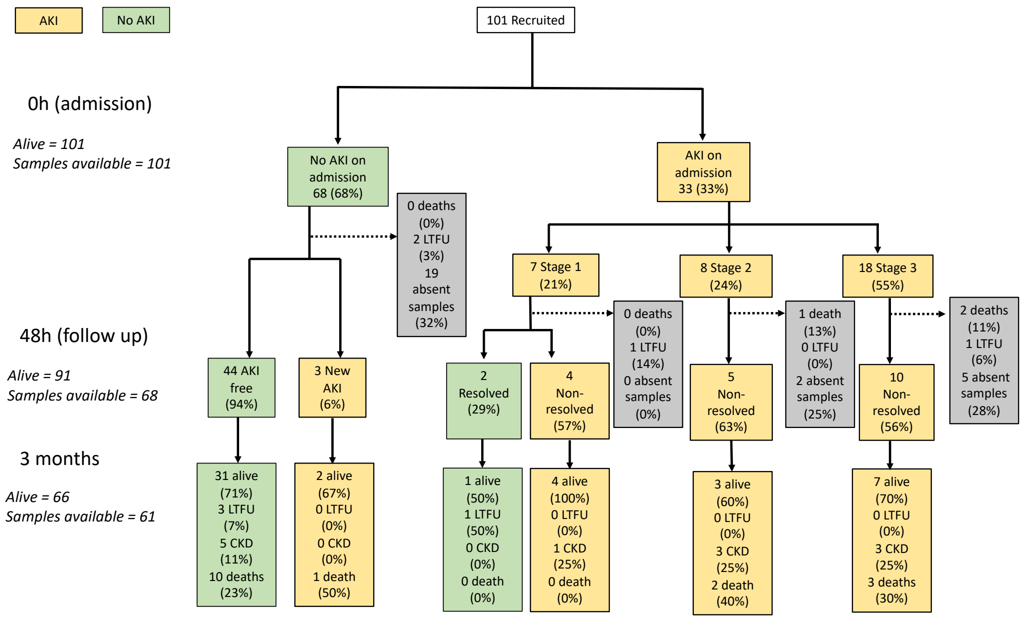

Supplementary figure 3: study flow diagram. Adults admitted to Zomba District Hospital with sepsis were recruited (n=101). AKI was defined using the KDIGO criteria based on a 1.5 fold increase in admission creatinine from estimated baseline, stratified by stages 1-3. AKI recovery was assessed at 48h and defined as recovered if either partial (creatinine within 50% of estimated baseline) or complete (creatinine returned to within 15% of estimated baseline). New (incident) AKI was assessed at 48h and diagnosed according to KDIGO using estimated baseline creatinine. CKD was assessed at 3-months and defined as an estimated glomerular filtration rate < 60 mL/min/1.73/m^2 according to MDRD equation. *LTFU* = lost to follow up.

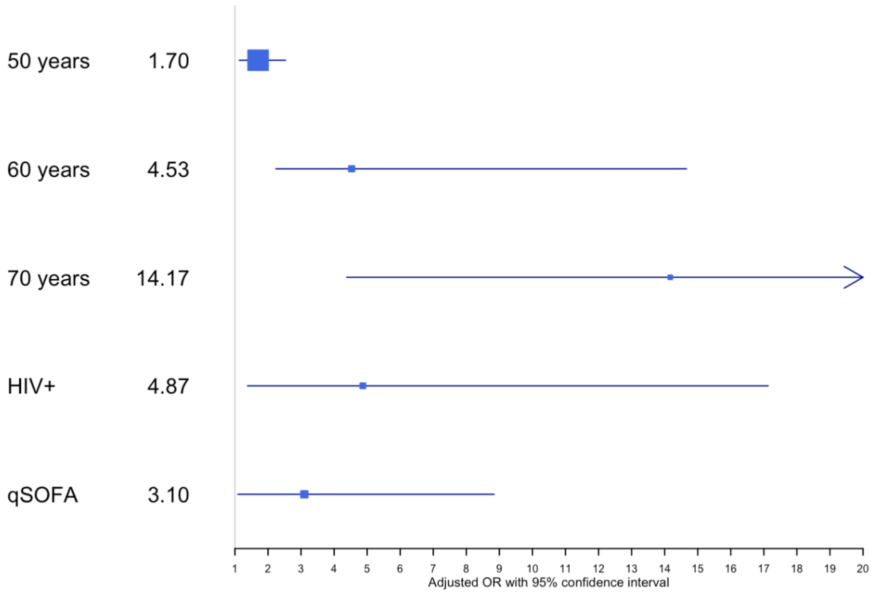

Supplementary figure 4: forest plot demonstrating AKI risk factors presented as odds ratios and 95% confidence intervals. Age categories are reported with respect to the reference category, 40 years. Number to the right of category = OR. Vertical line represents OR of 1.

For peer review only - http://bmjopen.bmj.com/site/about/guidelines.xhtml

a

HIV -ve	qSOFA score		
**Age (years)**	**1**	**2**	**3**
**20**	0.09	0.30	1.00
**40**	0.02	0.09	0.30
**60**	0.09	0.30	1.00
**80**	0.82	2.85	10.11

b

HIV +ve	qSOFA score		
**Age (years)**	**1**	**2**	**3**
**20**	0.45	1.50	5.25
**40**	0.12	0.43	1.50
**60**	0.45	1.50	5.25
**80**	4.26	13.29	49.00

Supplementary figure 5
a AKI odds for HIV negative participants with diastolic blood pressure held constant at 80 mm Hg
b AKI odds for people living with HIV with diastolic blood pressure held constant at 80 mm Hg. Colour coding yellow to orange depicts increasing odds.

For peer review only

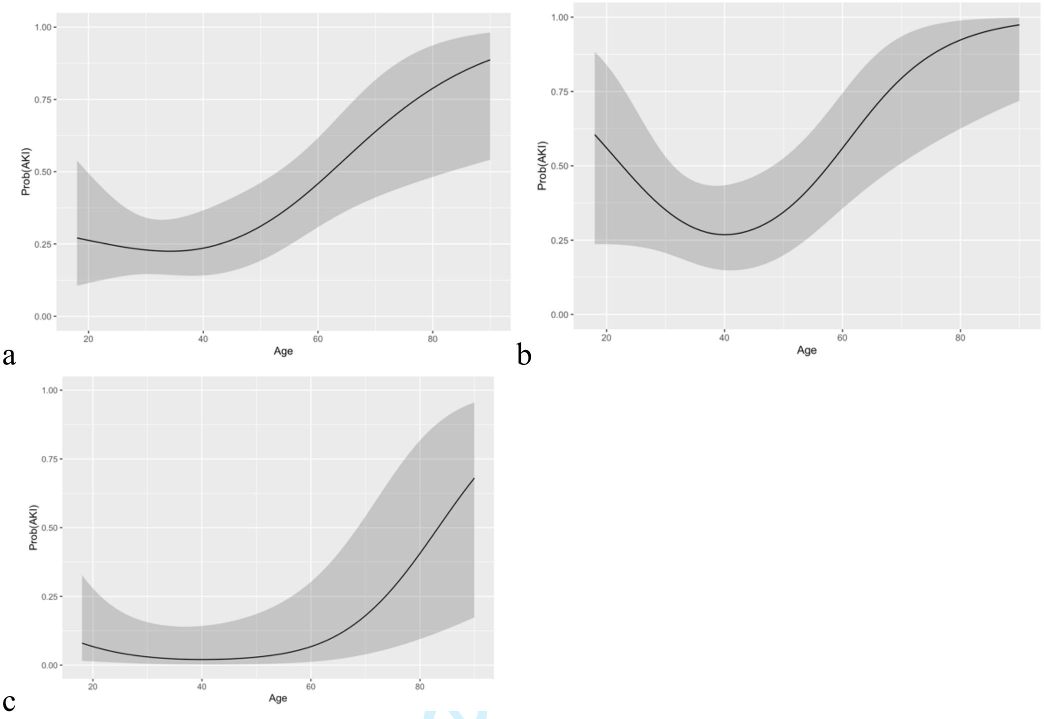

Supplementary figure 6
a unadjusted effect of age on probability of AKI. Age is expressed as a spline term with 3 knots.
b adjusted effect of age on probability of AKI. Diastolic blood pressure = 75 mm Hg, qSOFA score = 2, HIV status = positive.
c Adjusted effect of age on probability of AKI. Diastolic blood pressure = 75 mm Hg, qSOFA score = 1, HIV status = negative.

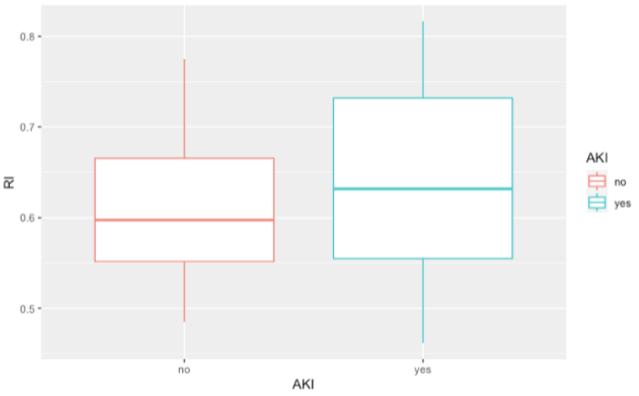

Supplementary figure 7: boxplot of RI distributions by AKI status
RI = renal resistive index.

For peer review only - http://bmjopen.bmj.com/site/about/guidelines.xhtml

Supplementary table 1: ultrasound, laboratory and derived indices.

Variable		Value
Ultrasound	Kidney Size (cm)	10 [9-10]
	Hydronephrosis*	16/100 (16%)
	Loss of corticomedullary differentiation*	44/100 (44%)
	Increased echogenicity†	41/99 (41%)
	Renal Resistive Index	0.62 [0.55-0.68]
Urine	Able to pass urine	97/101 (96%)
	Proteinuria on dipstick	29/101 (29%)
	Proteinuria (mg/dL)	0.0 [0.0-138.5]
	Microscopic Haematuria	54/101 [53]
Observations	Systolic blood pressure (mm Hg)	117 [103-133]
	Diastolic blood pressure (mm Hg)	75 [64-84]
	Heart rate (beats/min)	101 [84-123]
	Temperature (°C)	37 [37-38]
	Oxygen saturation (%)	98 [96-99]
	Respiratory rate (breaths/min)	24 [22-28]
	Glasgow coma score < 15	27/101 (27%)
Illness severity	Unable to stand unaided	27/101 (27%)
	Length of time unwell for (days)	7.0 [3.0-21.0]
	Receiving intravenous fluid	63/101 (62%)
	qSOFA score	2.0 [2.0-2.0]
Laboratory parameters	Haemoglobin (mmol/L)	11.9 [8.8-13.9]
	Blood Urea Nitrogen (mmol/L)	15 [8-30]
	Chloride (mmol/L)	104 [99-108]
	Sodium (mmol/L)	136 [131-140]
	Potassium (mmol/L)	3.6 [3.3-4.5]
	Bicarbonate (mmol/L)	23 [19-26]
	Creatinine (mmol/L)	0.80 [0.60-1.60]
	Anion Gap (mmol/L)	16 [15-18]

Values are median [interquartile range].
* Hydronephrosis/corticomedullary differentiation missing for 1 participant n=100
† Echogenicity missing n=2

For peer review only - http://bmjopen.bmj.com/site/about/guidelines.xhtml

Supplementary table 2: demographic univariable associations with AKI status.

Variable	No AKI	AKI	*p* value
**Age† (years)**	**35.75 [28.62-46.06]**	**46.46 [35.44-65.43]**	**0.01**
Male sex*	32 (47)	15 (45)	1.00
HIV infected*	33 (55)	20 (71)	0.29
Receiving antiretroviral therapy*	26 (79)	16 (84)	0.39
Receiving co-trimoxazole preventative therapy*	25 (76)	15 (79)	0.52
Receiving isoniazid preventative therapy*	6 (18)	5 (26)	0.33
Received TB treatment*	6 (9)	5 (15)	0.33
Prior Malaria*	12 (18)	9 (27)	0.30
Diagnosis of Diabetes*	0 (0)	1 (3)	0.33
Diagnosis of Hypertension*	2 (3)	5 (15)	0.04
Antibiotic use prior*	51 (75)	29 (88)	0.19
Over the counter/traditional meds*	2 (3)	2 (6)	0.60
Vomiting*	14 (21)	11 (33)	0.22
Diarrhoea*	10 (15)	6 (18)	0.77
Cough*	26 (38)	17 (52)	0.28

Values are median [interquartile range].
* Fisher's exact test † Wilcoxon rank sum

For peer review only - http://bmjopen.bmj.com/site/about/guidelines.xhtml

Supplementary table 3: ultrasound and urine related univariable associations with AKI status.

Variable	No AKI	AKI	*p* value
Kidney Size† (cm), median (IQR)	9.8 (8.96-10.49)	9.63 (8.67-10.35)	0.65
Hydronephrosis* n/N (%)	9 (13%)	7 (21%)	0.39
Loss of corticomedullary differentiation* n/N (%)	28 (42%)	16 (48%)	0.53
Increased echogenicity* n/N (%)	25 (38%)	16 (48%)	0.29
Renal Resistive Index†, median (IQR)	0.6 (0.55-0.66)	0.63 (0.56-0.73)	0.19
Able to pass urine* n/N (%)	67 (99%)	30 (91%)	0.10
**Proteinuria on dipstick* n/N (%)**	**11 (16%)**	**18 (55%)**	**<0.01**
**Proteinuria† (mg/dL), median (IQR)**	**0 (0-26.4)**	**182.1 (65.4-241.7)**	**<0.01**
**Microscopic Haematuria* n/N (%)**	**27 (40%)**	**27 (82%)**	**<0.01**

Values are median [interquartile range].
* Fisher's exact test † Wilcoxon rank sum

For peer review only - http://bmjopen.bmj.com/site/about/guidelines.xhtml

Supplementary table 4: observation and laboratory derived univariable associations according to AKI status.

Variable	No AKI	AKI	*p* value
Temperature† (°C)	37.2 [36.85-37.8]	37 [36.8-37.8]	0.58
Heart rate† (beats/min)	99.5 [83.75-124]	102 [92-123]	0.51
Respiratory rate† (breaths/min)	23.5 [20-26.25]	26 [23-29]	0.08
Systolic blood pressure† (mm Hg)	115.5 [103.75-128.25]	118 [101-141]	0.28
Diastolic blood pressure† (mm Hg)	74 [64-83.25]	77 [63-87]	0.35
Oxygen saturation† (%)	98 [96-99]	97 [95.5-98]	0.07
Glasgow coma score < 15*	17 (25)	10 (30)	0.63
Unable to stand unaided*	15 (22)	12 (36)	0.15
Length of time unwell† (days)	9 [3-30.44]	5 [3-14]	0.18
Receiving intravenous fluid*	38 (56)	25 (76)	0.08
qSOFA score†	2 [2-2]	2 [2-2]	0.27
Haemoglobin† (mmol/L)	11.9 [9.2-13.75]	11.55 [8.1-14.6]	0.88
**Blood Urea Nitrogen† (mmol/L)**	**10 [7-17.5]**	**40.5 [22.75-82.25]**	**<0.01**
Chloride† (mmol/L)	102.5 [98-108]	105.5 [101.75-109.75]	0.09
Sodium† (mmol/L)	136 [130.75-140]	136 [131-140]	0.99
Potassium† (mmol/L)	3.6 [3.3-4.2]	3.6 [3.17-5.18]	0.80
**Bicarbonate† (mmol/L)**	**24 [22-26]**	**16 [14-23]**	**<0.01**
**Creatinine† (mmol/L)**	**0.7 [0.5-0.8]**	**2.7 [1.6-6.3]**	**<0.01**
Anion Gap† (mmol/L)	16.5 [15-18]	16.5 [15-20]	0.24
**eGFR† (ml/min/1.73/m^2) (MDRD)**	**124.04 [92.68-159.04]**	**22.95 [11.21-40.24]**	**<0.01**
**Creatinine clearance† (mL/min) (Cockroft and Gault)**	**118.25 [93.71-143.71]**	**24.88 [14.48-33.66]**	**<0.01**
Chronic kidney disease at 3 months*	5 (12)	7 (37)	0.06

Values are median [interquartile range].
* Fisher's exact test † Wilcoxon rank sum
MDRD 4-parameter Modification of Diet in Renal Disease equation

For peer review only - http://bmjopen.bmj.com/site/about/guidelines.xhtml

Supplementary Table 5: complete case analysis for logistic regression analysis of risk factors associated with AKI.

Risk factor	Unadjusted OR (95% CI) single variable model [p value]	Adjusted OR (95% CI) Multivariable model [p value]
Age in years		
20	1.07 (0.25-4.26)	1.84 (0.53-39.50)
30	0.31 (0.52-1.61)	1.13 (0.70-3.90)
40 (ref)	1	1
50	**1.63 (1.14-2.21)**	**1.57 (1.03-2.49)**
60	**3.45 (1.60-7.20)**	**3.60 (1.77-14.45)**
70	**8.06 (2.24-30.72)**	**9.52 (2.95-134.78)**
80	**18.93 (3.14-137.70)**	**25.32 (4.91-1206.17)**
Sex - Male	0.95 (0.545-1.68) [0.88]	-
HT diagnosis	**2.40 (1.37-4.21) [<0.01]**	-
HIV positive	1.65 (0.82-3.32) [0.16]	**5.14 (1.47-22.25) [0.02]**
BMI	0.97 (0.90-1.04) [0.39]	-
DBP	**1.01 (1.00-1.02) [<0.01]**	1.03 (1.00-1.07) [0.05]
HCO3	**0.78 (0.69-0.86) [<0.01]**	-
SpO2	0.91 (0.77-1.00) [0.22]	-
Microscopic haematuria	**3.92 (1.77-8.66) [<0.01]**	-
Proteinuria	**2.98 (1.75-5.07) [<0.01]**	-
RI	**58.48 (1.32-2596.32) [0.04]**	-
Kidney size (cm)	0.85 (0.68-1.09) [0.21]	-
Loss of CMD	1.19 (0.69-2.09) [0.53]	-
Increased echogenicity	1.33 (0.77-2.32) [0.31]	-
qSOFA	1.31 (0.81-2.11) [0.28]	**3.43 (1.15-12.23) [0.04]**

Variables for inclusion in the full model selected based on hypothesised causal model (Supplementary Figure 1c).
HT hypertension *RI* resistive index *BP* blood pressure *HCO3* serum bicarbonate, *SpO2* capillary oxygen concentration *CMD* corticomedullary differentiation

For peer review only - http://bmjopen.bmj.com/site/about/guidelines.xhtml

STROBE cohort study checklist:

Risk factors for acute kidney injury and outcomes among sepsis admissions in Malawi.

Description	Item number	Recommendation	Present?	Where described
Title and abstract	1	(*a*) Indicate the study's design with a commonly used term in the title or the abstract	Yes	Abstract p2
		(*b*) Provide in the abstract an informative and balanced summary of what was done and what was found	Yes	Abstract p2
Introduction				
Background/rationale	2	Explain the scientific background and rationale for the investigation being reported	Yes	Introduction p3
Objectives	3	State specific objectives, including any prespecified hypotheses	Yes	Introduction p4
Methods				
Study design	4	Present key elements of study design early in the paper	Yes	Methods p4
Setting	5	Describe the setting, locations, and relevant dates, including periods of recruitment, exposure, follow-up, and data collection	Yes	Abstract p2<br>Methods p4-8
Participants	6	(*a*) Give the eligibility criteria, and the sources and methods of selection of participants. Describe methods of follow-up	Yes	Methods p4-6
		(*b*) For matched studies, give matching criteria and number of exposed and unexposed	NA	NA
Variables	7	Clearly define all outcomes, exposures, predictors, potential confounders, and effect modifiers. Give diagnostic criteria, if applicable	Yes	Methods p4-8<br>Supplement p1
Data sources/ measurement	8	For each variable of interest, give sources of data and details of methods of assessment (measurement). Describe comparability of assessment methods if there is more than one group	Yes	Methods p4-8
Bias	9	Describe any efforts to address potential sources of bias	Yes	Methods p6-8<br>Supplement p1
Study size	10	Explain how the study size was arrived at	Yes	Methods p8

For peer review only

Quantitative variables	11	Explain how quantitative variables were handled in the analyses. If applicable, describe which groupings were chosen and why	Yes	Methods p7
Results				
Participants	13	(a) Report numbers of individuals at each stage of study—eg numbers potentially eligible, examined for eligibility, confirmed eligible, included in the study, completing follow-up, and analysed	Yes	Supplementary figure 2, p2
		(b) Give reasons for non-participation at each stage	Yes	Supplementary figure 2, p2
		(c) Consider use of a flow diagram	Yes	Supplementary figure 2, p2
Descriptive data	14	(a) Give characteristics of study participants (eg demographic, clinical, social) and information on exposures and potential confounders	Yes	Results p8-9
		(b) Indicate number of participants with missing data for each variable of interest	Yes	Supplement p2
		(c) Summarise follow-up time (eg, average and total amount)	Yes	Results p8
Outcome data	15	Report numbers of outcome events or summary measures over time	Yes	Results p10 Supplementary figure 2, p2
Main results	16	(*a*) Give unadjusted estimates and, if applicable, confounder-adjusted estimates and their precision (eg, 95% confidence interval). Make clear which confounders were adjusted for and why they were included	Yes	Results p11 Supplement p10
		(*b*) Report category boundaries when continuous variables were categorized	NA	NA
		(*c*) If relevant, consider translating estimates of relative risk into absolute risk for a meaningful time period	NA	NA
Other analyses	17	Report other analyses done—eg analyses of subgroups and interactions, and sensitivity analyses	NA	Supplement p10
Discussion				
Key results	18	Summarise key results with reference to study objectives	Yes	Discussion p12

For peer review only

Limitations	19	Discuss limitations of the study, taking into account sources of potential bias or imprecision. Discuss both direction and magnitude of any potential bias	Yes	Discussion p13-14
Interpretation	20	Give a cautious overall interpretation of results considering objectives, limitations, multiplicity of analyses, results from similar studies, and other relevant evidence	Yes	Discussion p14
Generalisability	21	Discuss the generalisability (external validity) of the study results	Yes	Discussion p14
Other information				
Funding	22	Give the source of funding and the role of the funders for the present study and, if applicable, for the original study on which the present article is based	Yes	Funding p15

For peer review only

BMJ Open

Prospective cohort study to identify prevalence, risk factors and outcomes of infection associated kidney disease in a regional hospital in Malawi.

Journal:	*BMJ Open*
Manuscript ID	bmjopen-2022-065649.R1
Article Type:	Original research
Date Submitted by the Author:	21-Sep-2022
Complete List of Authors:	Carey, Laura; Liverpool School of Tropical Medicine, Clinical Sciences; Malawi-Liverpool-Wellcome Trust Clinical Research Programme,<br>Kaimba, Sylvester; Malawi-Liverpool-Wellcome Trust Clinical Research Programme<br>Nyirenda, Saulos; Zomba Central Hospital, Medicine<br>Chetcuti, Karen; Kamuzu University of Health Sciences<br>Joekes, Elizabeth; Liverpool School of Tropical Medicine, Clinical Sciences<br>Henrion, Marc; Malawi-Liverpool-Wellcome Trust Clinical Research Programme, ; Liverpool School of Tropical Medicine,<br>Rylance, Jamie; Liverpool School of Tropical Medicine, Clinical Sciences; Malawi-Liverpool-Wellcome Trust Clinical Research Programme,
**Primary Subject Heading**:	Renal medicine
Secondary Subject Heading:	Global health, Epidemiology, HIV/AIDS, Infectious diseases
Keywords:	Epidemiology < TROPICAL MEDICINE, Acute renal failure < NEPHROLOGY, Adult nephrology < NEPHROLOGY, Nephrology < INTERNAL MEDICINE

SCHOLARONE™
Manuscripts

I, the Submitting Author has the right to grant and does grant on behalf of all authors of the Work (as defined in the below author licence), an exclusive licence and/or a non-exclusive licence for contributions from authors who are: i) UK Crown employees; ii) where BMJ has agreed a CC-BY licence shall apply, and/or iii) in accordance with the terms applicable for US Federal Government officers or employees acting as part of their official duties; on a worldwide, perpetual, irrevocable, royalty-free basis to BMJ Publishing Group Ltd ("BMJ") its licensees and where the relevant Journal is co-owned by BMJ to the co-owners of the Journal, to publish the Work in this journal and any other BMJ products and to exploit all rights, as set out in our licence.

The Submitting Author accepts and understands that any supply made under these terms is made by BMJ to the Submitting Author unless you are acting as an employee on behalf of your employer or a postgraduate student of an affiliated institution which is paying any applicable article publishing charge ("APC") for Open Access articles. Where the Submitting Author wishes to make the Work available on an Open Access basis (and intends to pay the relevant APC), the terms of reuse of such Open Access shall be governed by a Creative Commons licence – details of these licences and which Creative Commons licence will apply to this Work are set out in our licence referred to above.

Other than as permitted in any relevant BMJ Author's Self Archiving Policies, I confirm this Work has not been accepted for publication elsewhere, is not being considered for publication elsewhere and does not duplicate material already published. I confirm all authors consent to publication of this Work and authorise the granting of this licence.

For peer review only

AUTHORS

Laura Carey*[1,2] Sylvester Kaimba[1], Saulos Nyirenda[3], Karen Chetcuti[4,5], Elizabeth Joekes[2,5], Marc Y. R. Henrion[1,2], Jamie Rylance[1,2]

1 Malawi Liverpool Wellcome Clinical Research Programme, Blantyre, PO Box 30096, Malawi

2 Department of Clinical Sciences, Liverpool School of Tropical Medicine, Liverpool, UK, L3 5QA

3 Zomba Central Hospital, Malawi Ministry of Health, Zomba, Malawi

4 Kamuzu University of Health Sciences, Private Bag 360, Chichiri, Blantyre 3

5 Worldwide Radiology, Liverpool, UK

***Corresponding author**

Dr. Laura Carey

Liverpool School of Tropical Medicine

Pembroke Place

Liverpool

L3 5QA

E: lcarey@mlw.mw

T: +447745279825

TITLE

Prospective cohort study to identify prevalence, risk factors and outcomes of infection associated kidney disease in a regional hospital in Malawi.

Word count: 4659

For peer review only - http://bmjopen.bmj.com/site/about/guidelines.xhtml

ABSTRACT

Objectives: Acute kidney injury (AKI) is a common and severe complication of community acquired infection, but data on impact in sub-Saharan Africa (SSA) are lacking. We determined prevalence, risk factors and outcomes of infection associated kidney disease in adults in Malawi.

Design: A prospective cohort study of adults admitted to hospital with infection, from Feb-Jun 2021, collecting demographic, clinical, laboratory and ultrasonography data.

Setting: Adults admitted to a regional hospital in Southern Region, Malawi.

Primary and secondary outcome measures: The primary outcomes were prevalence of kidney disease and mortality by Cox proportional hazard model. Acute kidney injury (AKI) was defined according to Kidney Disease Improving Global Outcomes (KDIGO) guidelines. Secondary outcomes were risk factors for AKI identified by logistic regression and prevalence of chronic kidney disease at 3 months.

Results: We recruited 101 patients presenting to hospital with infection. Median age was 38 years [IQR 29-48]), 88 had known HIV status of which 53 (60%) were living with HIV, and of these 42 (79%) were receiving antiretroviral therapy. AKI was present in 33/101 at baseline, of which 18/33 (55%) cases were severe (KDIGO stage 3). At 3 months 28/94 (30%) participants had died, while 7/61 (11%) of survivors had chronic kidney disease. AKI was associated with older age (age 60 versus 40: OR 3.88, 95% CI 1.82-16.64), and HIV positivity (OR 4.08, 95% CI 1.28-15.67). Living with HIV was independently associated with death (HR 3.97, 95% CI 1.07-14.69).

Conclusions: Kidney disease is common among hospitalized adults with infection in Malawi, with significant kidney impairment identified at 3 months. Our study highlights the difficulty in diagnosing acute and chronic kidney disease, and the need for more accurate methods than creatinine based eGFR equations for populations in

For peer review only - http://bmjopen.bmj.com/site/about/guidelines.xhtml

Africa. Patients with kidney impairment identified in hospital should be prioritised for follow up.

Word count: 300/300

For peer review only

For peer review only - http://bmjopen.bmj.com/site/about/guidelines.xhtml

Strengths and limitations of this study

- We provide data on prevalence, risk factors, and consequences of acute kidney injury (AKI), which are scarce in sub-Saharan Africa (SSA).
- We make use of the expanding availability of point of care ultrasound in Malawi and assess its role in the AKI assessment.
- This is the first AKI study in Malawi reporting outcomes at 3 months, identifying significant kidney impairment and likely undiagnosed chronic kidney disease (CKD).
- Diagnosis of AKI and the distinction between CKD is a challenge when baseline kidney function is unknown; pre-existing CKD may have been misclassified as AKI.
- Our estimate of baseline kidney disease may be lower than actual prevalence as eGFR equations and creatinine-based methods consistently underestimate kidney disease in SSA making our findings even more serious and highlights the difficulties in using standard definitions of kidney impairment in this context.

INTRODUCTION

Globally, 1.7 million people die from acute kidney injury (AKI) every year, 80% of deaths are in low and middle income countries [1]. Community acquired infections are a common cause of AKI in sub-Saharan Africa (SSA) [2]. In patients presenting to hospital with AKI in SSA, 10-70% have stage 3, requiring kidney replacement therapy, with an inpatient mortality of 44% [3,4]. The prevalence of AKI and CKD in community acquired infection in Malawi is unknown; no data exist for outcomes beyond hospital discharge.

The International Society of Nephrology (ISN) initiative aims that 'no one should die of untreated AKI in low resource regions by 2025' [5]. In SSA, access to kidney function testing is limited, contributing to delayed AKI diagnosis and potential progression to kidney failure. In the region, patients are typically younger than those

For peer review only - http://bmjopen.bmj.com/site/about/guidelines.xhtml

from high-income countries, and the majority will have no prior diagnosis or creatinine ascertainment. Available conventional estimators of baseline kidney function underestimate AKI incidence e.g. using the Modification of Diet in Renal Disease (MDRD) equation to back-calculate creatinine assuming an eGFR of 75 ml/L/1.73m^2 underestimates AKI incidence by > 50% [6].

Kidney ultrasound is standard practice in Malawi as part of the AKI assessment to identify chronic damage in the face of competition for dialysis beds. Patients with evidence of chronic kidney impairment are unlikely to be prioritized for kidney replacement therapy. In addition, kidney ultrasound may be used to identify those at increased risk of AKI, through assessment of kidney morphology, or by doppler estimation of the Renal Resistive Index (RI) which directly correlates with intra-renal arterial resistance.

A systematic review of 176 patients found that raised RI values on ultrasound were associated with increased odds of persistent AKI [7]. Given the expanding availability of ultrasound in Low- and Middle-Income Countries (LMICs), we assessed RI and other sonographic parameters in the diagnosis and risk-stratification of AKI in patients admitted to hospital with infection.

We aimed to estimate the prevalence of kidney disease at presentation and 3 month follow up in non-hospital-acquired infection in Malawian adults. Secondarily, we evaluated risk factors for kidney disease and outcomes of mortality.

METHODS

Setting

Zomba Central Hospital (ZCH) is a regional hospital in Southern Malawi serving both urban and rural populations with sixty adult medical inpatient beds. Healthcare is provided free at this government institution at the point of delivery. Approximately half of the patients usually present directly to the hospital and the other half are

For peer review only - http://bmjopen.bmj.com/site/about/guidelines.xhtml

referred from primary or secondary care. Adults are triaged by a nurse and then reviewed by a doctor or clinical officer. If admission under a specialty team is deemed appropriate, they will be reviewed by an intern or registrar from the admitting speciality, and by a consultant usually within 24 hours.

Limited medical investigations are available on clinician request. Depending on availability, standard of care includes TB diagnostics (sputum smear microscopy, Xpert/MTB/Rif, urine lipoarabinomannan), malaria testing (usually rapid diagnostic test), plain film radiography, full blood counts and serum creatinine ascertainment. Inpatient HIV services include provider-initiated HIV testing and counselling, with CD4 count and viral load available on clinician request. A 4-bed enhanced observation unit exists on the medical ward with provisions for oxygen therapy, but no cardiac monitoring or defibrillator. There is a 5-bed intensive care unit, mostly used for surgical post operative patients. Local HIV prevalence is estimated at 17% among 15-49 year olds [8].

For peer review only

At ZCH, we performed a prospective observational study recruiting adults (≥ 18 years) with suspected infection and a medical decision to admit to hospital. Suspected infection was deliberately broad to incorporate a wide spectrum of presenting physiology, and to be consistent with ongoing studies. This includes reporting in the last week any of: fever; rigors; night sweats; treatment with antibiotics. All patients in the admissions unit were approached for screening, Monday-Friday, 0700-1700.

Exclusion criteria were any of: people lacking capacity to consent, with no proxy consent available; pregnancy; non-severe uncomplicated localised infection with only local symptoms apparent on clinical examination by the screening clinical officer (such as cellulitis); detainees; trauma; admission to surgical or obstetric/gynaecological ward; previously recruited to the study. Responsibility for care and treatment decisions remained with the usual clinical team. Participants were followed-up as inpatients at 48 hours, and at 3 months in the community.

Sampling and Laboratory methods

For peer review only - http://bmjopen.bmj.com/site/about/guidelines.xhtml

Patients provided blood and urine at baseline, 48 hours, and 3 months. Creatinine, blood urea nitrogen (BUN), glucose, ionized calcium, sodium, potassium, chloride, and bicarbonate were assessed by iSTAT CHEM8 cartridges (Abbott Point of Care Inc., Princeton, NJ). The handheld iSTAT system measures creatinine using an enzymatic assay traceable to the U.S NIST Standard Reference material SRM909 with a reportable range of 0.20-20.0 mg/dL. Standard of care for measurement of serum creatinine in ZCH is using a sarcosine oxidase (enzymatic) method on a Mindray BS360-E machine. Often this test is unavailable due to reagent stock outs which are supplied by the government.

Point of care HIV testing was done for those with unknown status or no recent negative test. Urine was tested by dipstick for blood and protein, and positive samples further quantified using a laboratory analyser (Mindray BS360).

Ultrasound

Point of care ultrasound assessed kidney size, corticomedullary differentiation, echogenicity, and presence of any hydronephrosis defined as a dilated appearance of the collecting system. Assessment was made by a clinical officer who underwent dedicated training, including direct supervision by a consultant radiologist before study commencement.

RI was measured at each pole of each kidney (upper, middle, lower) and an average taken across poles and between kidneys giving one RI value per participant. A portable Mindray DP-50 machine with 5MHz convex probe was used throughout. The assessor was blinded to the kidney diagnosis.

AKI assessment

Creatinine was measured on admission, at 48 hours and at 3 month follow up.
AKI was defined according to KDIGO as a 1.5 fold increase in baseline creatinine from the estimated baseline [9]. AKI severity as fold increase over estimated baseline was defined according to KDIGO as: stage 1 (1.5-1.9x); stage 2 (2.0-2.9x); stage 3 (≥3.0x).

For peer review only - http://bmjopen.bmj.com/site/about/guidelines.xhtml

We refer to incident AKI at 48 hours if AKI criteria were not met at baseline, but there was a creatinine rise within 48 hours. AKI recovery was defined as a decrease of 0.3 mg/dL between baseline and 48 hours or a normal creatinine (< 1.3 mg/dL) at 3 months.

Acute kidney disease (AKD) was assessed on admission and at 3 months and defined according to KDIGO as: under 3 month duration, GFR < 60 ml/min/1.73m^2 or decrease in GFR by ≥ 35% or increase in creatinine by > 50% and/or albuminuria/haematuria [10]. Urine output was not assessed as it is not universal clinical practice in Malawi.

AKI associated with infection, particularly in LMICs, affects patients unlikely to have a documented baseline creatinine, necessitating estimation through back-calculation [6]. No participants had a previous documented creatinine result. Given the young age distribution of the population with relatively few comorbidities, baseline creatinine was estimated using a Modification of Diet in Renal Disease (MDRD) equation, assuming a glomerular filtration rate (GFR) of 100 mL/min [6]. MDRD was modified to exclude the race coefficient as eGFR equations using ethnicity adjustment factors overestimate GFR, leading to underdiagnosis of CKD and negative bias in people of Black ethnicity [11–13].

The full-age spectrum equation was used to back calculate an estimate of baseline creatinine: using MDRD may be imprecise in estimating baseline creatinine in older adults, resulting in lower baseline estimates and more AKI diagnosis at older ages [14]. In addition, we estimated AKI using lowest creatinine during admission as a baseline value. A sensitivity analysis compared the effect of each method of baseline creatinine estimation on AKI incidence.

Illness severity

Illness severity was measured by quick sepsis related organ failure assessment score (qSOFA) which uses three criteria: one point for low blood pressure (systolic blood

For peer review only - http://bmjopen.bmj.com/site/about/guidelines.xhtml

pressure ≤ 100 mm Hg), high respiratory rate (≥ 22 breaths per minute), or altered mental state (Glasgow coma scale < 15).

Mortality and CKD

Mortality was defined as death by 48 hours or 3 months. CKD was assessed at 3 months and defined as an eGFR of < 60 mL/min/1.73m^2 calculated using combined results from the Chronic Kidney Disease Epidemiology Collaboration (CKD-EPI) and the 4-parameter Modification of Diet in Renal Disease (MDRD) study equation, for a non-IDMS-traceable creatinine method, without race adjustment [15–17]. GFR estimates were reported as adjusted for body surface area [15]. Those with an eGFR > 60 mL/min/1.73m^2 at presentation were excluded from the CKD definition as the 3 month duration had not been proven. Those with an eGFR < 60 mL/min/1.73m^2 at presentation and 3 month follow up were given a diagnosis of CKD.

Statistical Analysis

Statistical analyses were performed in R (v 4.0.2) [16,18]. Data and code are publicly available in a GitHub repository [19]. Summary statistics were calculated for the cohort which was then divided into AKI and no-AKI groups, described using median and interquartile range (IQR) for continuous variables and proportions for categorical variables. Two sample t-tests or non-parametric tests, depending on data distribution, were used to compare variables between groups, and Fisher's test for proportions. All methods and reporting follow the Strengthening and Reporting of Observational Studies in Epidemiology guidelines [20].

A directed acyclic graph was used to examine the hypothesized causal relationship between HIV exposure and AKI (supplementary figure 1), and to identify any possible confounding. Age was identified as a confounder, and admission to hospital a collider, as participants were conditioned on this variable by inclusion in the study. Risk factors for AKI were considered and used for logistic regression analysis. Logistic regression outputs were reported as odds ratio (OR) and 95% confidence intervals.

For peer review only - http://bmjopen.bmj.com/site/about/guidelines.xhtml

Age was included in the adjusted model as a cubic spline term with 3 knots, meaning that we used age as a continuous variable rather than categorising it into a limited set of age bands. The effect of age on probability of AKI was modelled using predicted probabilities from the adjusted model (when qSOFA score = 2, HIV status = positive, and diastolic blood pressure = 75 mm Hg). Odds ratios for different ages were generated by comparing odds to the reference age, chosen here to be 40 years [21]. Bootstrap sampling and the percentile method with 1,000 replicates were then used to construct 95% confidence intervals for the odds ratio.

Kaplan-Meier survival analysis was conducted using Cox proportional hazards adjusted for age and qSOFA score.

Sample Size

A power calculation for specific risk factors was not performed before initiation of this exploratory study. However, a post-hoc power calculation was performed in G Power (v 3.1), given the achieved sample size (88 participants with recorded HIV and AKI status), using HIV as the risk factor [22]. Given the observed prevalence of AKI in HIV negative participants at 23% (8/35), with HIV prevalence at 60% (53/88), for a two tailed test, with 80% power and 0.05 alpha, the study was powered to detect effect sizes with an OR at least as large as 3.79.

Missing data

Missing data was less than 18% across all variables (supplementary figure 2) so results of a complete case analysis are reported. The main reason for missing follow up data was participants being uncontactable, or unable to get to ZCH for a follow up blood test due to transport or logistical reasons.

Patient and public involvement

At the end of the study period, participants, patients, and staff at ZCH were invited to a kidney awareness day, where key results were disseminated using simple leaflets, presentations, and a group discussion.

For peer review only - http://bmjopen.bmj.com/site/about/guidelines.xhtml

RESULTS

Between 3rd February 2021 and 19th July 2021, 101 eligible patients admitted with infection were recruited. The study flow diagram is summarised in supplementary figure 3. Table 1 summarises the baseline characteristics of the participants. There were 47/101 (47%) male participants. The median age was 38 (interquartile range [IQR] 29-48). Median follow up time was 92 days (IQR 71-95). Median number of days unwell prior to hospital presentation was 7 (IQR 3-21).

Table 1: characteristics of participants.

Variable		Value
Demographics	Age (years)	38 [29-48]
	Male sex	47/101 (47)
	Body mass index (kg m^{-2})	22 [19-23]
HIV/TB/Malaria	HIV infected*, n (%)	53/88 (60%)
	Receiving antiretroviral therapy, n (%)	42/53 (79%)
	Receiving co-trimoxazole preventative therapy, n (%)	40/53 (75%)
	Receiving isoniazid preventative therapy, n (%)	11/53 (21%)
	History of receiving TB treatment, n (%)	11/101 (11%)
	Prior malaria, n (%)	21/101 (21%)
Comorbidities	Pre-existing diagnosis of diabetes mellitus, n (%)	1/101 (1%)
	Pre-existing diagnosis of hypertension, n (%)	7/101 (7%)
Drugs	Antibiotic use prior, n (%)	80/101 (79%)
	Traditional/over the counter medications, n (%)	4/101 (4%)
Symptoms	Vomiting, n (%)	25/101 (25%)
	Diarrhoea, n (%)	16/101 (15%)
	Cough, n (%)	43/101 (43%)

Values are median [interquartile range].
* HIV status missing for 16 participants

For those who HIV status was known (88/101), 53/88 (60%), of participants were people living with HIV. Among people living with HIV, 42/53 (79%) were receiving

For peer review only - http://bmjopen.bmj.com/site/about/guidelines.xhtml

antiretroviral therapy, 40/53 (75%) co-trimoxazole preventive therapy, and 11/53 (21%) isoniazid preventive therapy. Among all participants recruited, 11/101 (11%) had a history of receiving treatment for tuberculosis and 21/101 (21%) for malaria within the past month. The majority, 80/101 (79%), reported antibiotic use prior to presentation and 4/101 (4%) reported using over the counter medications or traditional medicines. No participants had a known prior diagnosis of CKD, 1/101 (1%) reported a history of diabetes, and 7/101 (7%) a history of hypertension.

A histogram of baseline creatinine values in mg/dL is presented in supplementary figure 4. Ultrasound, laboratory, and derived indices are summarised in supplementary table 1. Participants were classified according to AKI status. Using assumed GFR of 75 and 100 mL/min to estimate baseline creatinine, 28 (28%) and 33 (33%) respectively met the definition for AKI at presentation. Back calculation of baseline creatinine using the full age spectrum equation identified 36/101 (36%) cases of AKI, whereas using lowest creatinine value identified 17/101 (17%) cases of AKI. Overall, the majority of patients (63/101, 62%) met our definition of AKD at presentation.

Outcomes, and the effect of different methods to estimate baseline creatinine on AKI prevalence are presented in table 2. The prevalence of CKD and AKD among survivors with creatinine values available at 3 months was 7/61 (11%), and 11/61 (18%) respectively. Mortality among those contactable at 3 months (94/101) was 28/94 (30%).

For peer review only

Table 2: Outcomes

Timepoint		Value
0 hours	Creatinine mg/dL	0.80 (0.60-1.60)
	eGFR (mL/min/1.73/m^2) MDRD	93 [41-141]
	Creatinine clearance (mL/min) (Cockroft and Gault)	94 [35-129]
	Acute kidney injury (assumed GFR 100 mL/min) n (%)	33/101 (33%)
	Acute kidney injury (assumed GFR 75 mL/min), n (%)	28/101 (28%)
	Acute kidney injury (full age spectrum equation), n (%)	36/101 (36%)
	Acute kidney injury using lowest creatinine as baseline, n (%)	17/101 (17%)
	Acute kidney disease, n (%)	63/101 (62%)
	Stage 3 AKI, n (%)	18/33 (55%)
	Stage 2 AKI, n (%)	8/33 (24%)
	Stage 1 AKI, n (%)	7/33 (21%)
48 hours	Incident AKI§, n (%)	3/80 (4%)
	Recovered AKI, n (%)	18/33 (55%)
	Recovered acute kidney disease, n (%)	38/63 (60%)
	Alive at 48 hours‡, n (%)	91/94 (97%)
3 months	Alive at 3 months‡, n (%)	66/94 (70%)
	Acute kidney disease¶, n (%)	11/61 (18%)
	Chronic kidney disease (eGFR < 60 mL/min/1.73m^2 at baseline and 3 months)¶, n (%)	7/61 (11%)

Values are median [interquartile range].
§ 48h creatinine missing n = 21
‡ Lost to follow up n = 7
¶ 3 month creatinine missing in survivors n = 5

For peer review only - http://bmjopen.bmj.com/site/about/guidelines.xhtml

Odds ratios and 95% confidence intervals for AKI			
**Characteristic**	**Category**	**AKI OR (95% CI)**	
		**Unadjusted**	**Adjusted for age and HIV status**
Age	20	1.07 (0.25-4.26)	1.81 (0.86-22.19)
	30	0.91 (0.52-1.61)	0.91 (0.66-2.18)
	40 (ref)	1.00	1.00
	50	1.63 (1.14-2.21)	1.59 (1.14-2.46)_
	60	3.45 (1.60-7.20)	3.88 (1.84-16.64)
	70	8.07 (2.24-30.72)	14.59 (3.96-278.02)
	80	18.93 (3.14-137.70)	62.02 (9.82-5185.71)
Sex	Female (ref)	1.00	1.00
	Male	0.96 (0.55-1.68)	1.04 (0.38-2.82)
BMI (kg m^{-2})		0.97 (0.90-1.04)	0.92 (0.79-1.05)
HIV	Positive	1.65 (0.82-3.32)	4.08 (1.28-15.67)
	Negative	1.00	1.00

Demographic univariable associations with AKI status are summarised in supplementary table 2. Ultrasound and urine related univariable associations are presented in supplementary table 3. There was no significant difference in kidney appearances on ultrasound and renal resistive index between participants with AKI compared to those without AKI. Observation and laboratory univariable associations are in supplementary table 4 and the effect of age on AKI when the full age spectrum equation is used to back calculate creatinine is presented in supplementary table 5. Supplementary table 6 shows the effect of each definition of AKI and AKD on the mortality outcome: death by 3 months.

Multivariable associations with AKI status (based on estimated baseline creatinine assuming GFR 100 mL/min) identified by logistic regression are presented as unadjusted and adjusted ORs and 95% confidence intervals (CIs) in table 3.

For peer review only - http://bmjopen.bmj.com/site/about/guidelines.xhtml

Comorbidity	Hypertension	2.40 (1.37-4.21)	3.19 (0.43-29.27)
Structural	Microscopic haematuria	3.92 (1.77-8.66)	6.34 (1.99-24.87)
	Proteinuria	2.98 (1.75-5.07)	4.05 (1.39-12.22)
	Kidney size	0.86 (0.68-1.09)	1.32 (0.82-2.15)
	Increased echogenicity	1.33 (0.77-2.32)	1.61 (0.59-4.44)
	Loss of corticomedullary differentiation	1.20 (0.69-2.09)	0.99 (0.36-2.73)
	Resistive index	58.48 (1.32-2596.32)	70.11 (0.79-84185.95)
Physiological	Quick sequential organ failure assessment score (qSOFA)	1.31 (0.81-2.11)	1.79 (00.76-4.45)
	Oxygen saturations	0.91 (0.77-1.00)	0.98 (0.81-1.21)
	Diastolic blood pressure*	1.01 (1.01-1.02)	1.02 (0.99-1.05)
	Bicarbonate	0.78 (0.69-0.86)	0.79 (0.68-0.90)

Table 3: **Logistic regression analysis of risk factors associated with AKI**. Parameter estimates expressed as unadjusted and adjusted ORs and 95% confidence intervals. *Higher diastolic blood pressures were associated with AKI in the unadjusted model.

Increasing age, living with HIV, microscopic haematuria and proteinuria were most associated with AKI on logistic regression analysis. Supplementary figure 5 shows the odds of AKI for different ages, qSOFA scores and HIV status, with systolic blood pressure held constant at 80 mm Hg. Supplementary figure 6 presents the effect of age on probability of AKI, and supplementary figure 7 presents the RI distributions by AKI status as a boxplot.

For peer review only - http://bmjopen.bmj.com/site/about/guidelines.xhtml

Time-to-event analysis, adjusted for age and qSOFA score demonstrated an increased hazard of death for HIV positive participants (HR 3.97, 95% CI 1.07-14.69) (Figure 1 Kaplan-Meier curves).

DISCUSSION

Adults presenting with infection in Zomba, Malawi are young, and predominantly living with HIV. Prevalence of AKI depended on the method of defining baseline creatinine, but was between 17-36%, with that of AKD at 62%. The majority of AKI was severe (stage 3), and mortality at 3 months was 28/94 (30%). Living with HIV and increasing age were risk factors for presentation to hospital with AKI, and there was a significant prevalence of kidney disease at 3 months (11% CKD, 18% AKD). Living with HIV was independently associated with death at 3 months.

Other limited studies examining kidney impairment in Blantyre, Malawi found 21% of medical admissions had evidence of kidney disease on admission (17% AKI, and 3% AKD), which is lower than our reported prevalence for both, particularly AKD [4]. Of the Blantyre participants with AKI, 60% were stage 3 AKI, and inpatient mortality was 44% in patients with AKI compared to 14% with no kidney disease [4]. The mortality in patients with AKI in Blantyre is likely higher than ours in Zomba (44% versus 30%), as it included AKI in the context of general medical problems: malignancy, liver disease, heart failure and stroke, rather than AKI in the context of infection.

Worldwide, the incidence of AKI is not well known. One report of 312 studies including > 3.5 million patients mostly from hospitals in high-income countries suggests a pooled incidence of 22% (95% CI 19-24) and a mortality rate of 23% [23]. In high-income country intensive care settings, dialysis requiring AKI mortality rates can vary between 40-60% [24–26]. Organ dysfunction was not a requirement for inclusion in our study which may explain why our mortality is relatively lower.

For peer review only - http://bmjopen.bmj.com/site/about/guidelines.xhtml

To our knowledge, this is the first AKI study in Malawi to obtain creatinine values at 3 months post discharge. Our data suggest a CKD prevalence of 7/61 (11%). How our data compares to the true community population prevalence of CKD in Malawi is not known. Normative ranges for GFR have not been established in African populations, including whether a cut-off of GFR of less than 60 mL/min/1.73 m^2 is an appropriate definition of CKD [27].

In a recently published cohort, the African Research on Kidney Disease (ARK) study, in populations from Malawi, Uganda and South Africa, the prevalence of measured GFR < 60 mL/min/1.73 m^2 using iohexol plasma clearance was 19%. This is nearer our estimate of AKD prevalence at 3 months, than our estimate of CKD [27]. A cross-sectional survey of urban and rural Malawi using the CKD-EPI equation suggested a prevalence of eGFR < 60 mL/min/1.73m^2 of 1% , and a systematic review of 13 countries in SSA estimated the pooled prevalence of CKD to be 14% [28,29]. Prevalence reported by eGFR equations rather than measured GFR are likely to be significant underestimates, as highlighted by the important findings from the ARK study. Estimating GFR using serum creatinine substantially underestimates the population level burden of impaired kidney function in Africa [27]. AKD assessment, which is broader and includes albuminuria and haematuria, may be more representative of the true burden. Regardless, we urgently need more accurate methods to report kidney disease for the region, or risk continued underreporting.

An additional complication is that patients presenting with infections in Malawi do not have documented baseline creatinine values, which can be addressed by back-calculation estimation by GFR equations. However, the optimal equation to accurately estimate GFR in Malawi (and across SSA) is uncertain. In studies from SSA, coefficients for African-American ethnicity consistently overestimate GFR [30]. Similarly, applying creatinine based eGFR formulae in HIV without adjusting for BMI tends to overestimate GFR and therefore underestimate CKD burden [12].

We compared the different equations for estimating baseline creatinine and prevalence of AKI. Using lowest creatine during admission as a baseline identified fewer cases of

For peer review only - http://bmjopen.bmj.com/site/about/guidelines.xhtml

AKI (17%) than the MDRD and full age spectrum back calculation equations (28-36%), but may have been less likely to misclassify CKD as AKI. Comparatively, AKD encompasses a much broader definition and identified many more cases of kidney impairment (62%). Mortality at 3 months was similar for each AKI definition except lowest creatinine as baseline, for which there were no deaths, likely because this definition excluded participants with the most severe, but unchanged kidney impairment.

In this context, the inherent limitations of creatinine based AKI definitions are likely to substantially underestimate actual prevalence. Despite using high-income country based 'normal' eGFR values, this study still shows a high proportion of likely baseline kidney disease. Even if a substantial proportion of the baseline AKI was in fact undiagnosed CKD, this still represents a significant burden of kidney disease in adults in Malawi who present to hospital with infections. Furthermore, the binary AKI/CKD definitions are difficult, and dichotomisation may not even be meaningful or helpful in this context.

We did not find ultrasound to be useful in identifying patients at risk of AKI in our cohort. Furthermore, resistive indices can be technically challenging to obtain and are not commonly practiced in Malawi, and we note the wide confidence interval around our OR estimates. Our data does not support use of the RI in the AKI assessment and further investigation, with larger datasets, is needed. We suggest point-of-care ultrasound use as part of the AKI assessment, but not as a surrogate for serum creatinine measurement.

Most deaths occurred after discharge rather than in hospital, indicating long term mortality associated with hospitalisation with infection. Combined with the high prevalence of kidney disease identified at 3 months, community follow up of patients is essential. To reduce post-discharge deaths, patients should be counselled on avoiding nephrotoxic medications which are commonly used (for example non-steroidal anti-inflammatories), and advice given on how to prevent future episodes of acute kidney disease. Due to limited resources, follow up should be prioritized for

For peer review only - http://bmjopen.bmj.com/site/about/guidelines.xhtml

those identified with kidney impairment. Outpatient review after discharge should include assessment for recovery of kidney impairment, new acute kidney disease or formal diagnosis of CKD. Those identified with AKD/CKD will need ongoing follow up for CKD assessment and management to prevent progression to kidney failure.

Our study has several limitations. We do not know the true prevalence of CKD at presentation, given there were no historical serum creatinine results and patients did not report known CKD. This may have resulted in misclassification of CKD as AKI and AKD. It is reasonable to assume that the 7 cases of CKD (eGFR < 60 mL/min/1.73m^2 at both presentation and 3 month follow up) were misclassified as both AKD and AKI at presentation. Evidence of recovery at 3 months was seen in 18/33 (55%) of those with AKI and 38/63 (60%) with AKD, suggesting true AKI/AKD rather than misclassified CKD.

We were unable to identify incident CKD in those who had normal kidney function (eGFR > 60 mL/min/1.73m^2) at presentation at follow up as the 3-month rule would not be observed. However, there was frequent AKD at 3 months; 18% new kidney impairment in patients who had normal kidney function on admission. This study also highlights the significant prevalence of undiagnosed CKD (eGFR < 60 mL/min/1.73m^2 at both presentation and 3 month follow up), of which none of the participants had prior knowledge. The high prevalence of HIV in the cohort increases the likelihood of baseline kidney disease, and indeed the majority of the CKD at 3 months was in people living with HIV (5/7 [71%]). Estimates for CKD prevalence in people living with HIV across Africa are between 1-46% with a pooled prevalence of 6% [31].

The other significant limitation is related to there being no validated method to estimate GFR and baseline creatinine for Africans, in health or acute illness. We estimated baseline creatinine using an assumed eGFR of 100 mL/min, which resulted in a 5% difference in AKI prevalence compared to an assumed eGFR of 75 mL/min. An assumed eGFR between 75-100 mL/min is supported by the median (IQR) iohexol measured GFR in Malawians reported in the ARK study: 86 (72-100) mL/min/1.73m^2 and 92 (77-104) for women and men respectively [27].

For peer review only - http://bmjopen.bmj.com/site/about/guidelines.xhtml

We used MDRD and CKD-EPI equations to report eGFR normalised to 1.73 m^2 body surface area, however adults in Malawi may have a smaller body surface area. The median (IQR) body surface area for Malawian females and males in the ARK study was 1.70 m^2 (1.50-1.80) and 1.70 m^2 (1.60-1.80) respectively [27]. Urine output was not used to define AKI which may have led to underestimation and imprecision of AKI staging. Partial pressure of carbon dioxide (PCO_2) was not measured, preventing correction of iSTAT measurements for PCO_2. It was not possible to ascertain causes of death, and our study was not powered to detect moderate or small associations.

To prevent deaths from untreated AKI in low resource regions by 2025, diagnosis of AKI and kidney disease needs to be appropriate for the local context. We urgently need more accurate methods to assess kidney function for specific African populations. eGFR and creatinine based equations underreport the burden of kidney disease and the distinction between AKI and CKD is difficult [1]. Interventions to reduce the impact of kidney disease in Malawi should focus resources on identifying patients with kidney impairment for follow up to prevent progression to kidney failure. This is essential given the scarcity of kidney replacement therapy.

FUNDING

LC was funded by an institutional translational partnership award [grant 219633/Z/19/Z] from the Wellcome Trust to Malawi-Liverpool-Wellcome Trust Clinical Research Programme (MLW). JR is funded by a Wellcome Career Development Fellowship [grant 206545/Z/17/Z] and by the National Institute for Health Research (NIHR) (17/63/42) using UK aid from the UK Government to support global health research. The views expressed are those of the authors and not necessarily those of the National Health Service, NIHR or the Department of Health and Social Care. For the purpose of open access, the author has applied a CC BY public copyright licence to any Author Accepted Manuscript version arising from this submission.

For peer review only - http://bmjopen.bmj.com/site/about/guidelines.xhtml

COMPETING INTERESTS

The authors declare no competing interests.

AUTHOR CONTRIBUTIONS

LC and JR conceived and designed the study. SN was involved in the conceptualization and project administration. EJ and KC were involved in conceptualisation and point of care ultrasound and KC delivered the ultrasound training. SK collected the data and performed the ultrasounds. LC analysed the data and wrote the manuscript. MYRH advised on statistics. All authors read, commented on, and approved the final manuscript.

DATA AVAILABILITY

Data and code available at https://github.com/careyla/PARIS [19].

ETHICAL APPROVAL

Participants gave written informed consent under ethical approvals from the College of Medicine Research Ethics Committee, University of Malawi (P.03/19/2625) and the Liverpool School of Tropical Medicine Ethics Committee (18-062). Study information including purposes, benefits and risk was provided to all participants in both English and Chichewa.

REFERENCES

1 Mehta RL, Cerdá J, Burdmann EA, *et al.* International Society of Nephrology's 0by25 initiative for acute kidney injury (zero preventable deaths by 2025): a human rights case for nephrology. *Lancet Lond Engl* 2015;**385**:2616–43. doi:10.1016/S0140-6736(15)60126-X

For peer review only - http://bmjopen.bmj.com/site/about/guidelines.xhtml

2 Singer M, Deutschman CS, Seymour CW, *et al.* The Third International Consensus Definitions for Sepsis and Septic Shock (Sepsis-3). *JAMA* 2016;**315**:801–10. doi:10.1001/jama.2016.0287

3 Olowu WA, Niang A, Osafo C, *et al.* Outcomes of acute kidney injury in children and adults in sub-Saharan Africa: a systematic review. *Lancet Glob Health* 2016;**4**:e242-250. doi:10.1016/S2214-109X(15)00322-8

4 Evans RDR, Hemmilä U, Craik A, *et al.* Incidence, aetiology and outcome of community-acquired acute kidney injury in medical admissions in Malawi. *BMC Nephrol* 2017;**18**:21. doi:10.1186/s12882-017-0446-4

5 Mehta RL, Burdmann EA, Cerdá J, *et al.* Recognition and management of acute kidney injury in the International Society of Nephrology 0by25 Global Snapshot: a multinational cross-sectional study. *Lancet Lond Engl* 2016;**387**:2017–25. doi:10.1016/S0140-6736(16)30240-9

6 Cooper DJ, Plewes K, Grigg MJ, *et al.* An Evaluation of Commonly Used Surrogate Baseline Creatinine Values to Classify AKI During Acute Infection. *Kidney Int Rep* 2021;**6**:645–56. doi:10.1016/j.ekir.2020.12.020

7 Ninet S, Schnell D, Dewitte A, *et al.* Doppler-based renal resistive index for prediction of renal dysfunction reversibility: A systematic review and meta-analysis. *J Crit Care* 2015;**30**:629–35. doi:10.1016/j.jcrc.2015.02.008

8 Gupta-Wright A, Corbett EL, Oosterhout JJ van, *et al.* Rapid urine-based screening for tuberculosis in HIV-positive patients admitted to hospital in Africa (STAMP): a pragmatic, multicentre, parallel-group, double-blind, randomised controlled trial. *The Lancet* 2018;**392**:292–301. doi:10.1016/S0140-6736(18)31267-4

9 Khwaja A. KDIGO clinical practice guidelines for acute kidney injury. *Nephron Clin Pract* 2012;**120**:c179-184. doi:10.1159/000339789

10 Lameire NH, Levin A, Kellum JA, *et al.* Harmonizing acute and chronic kidney disease definition and classification: report of a Kidney Disease: Improving Global Outcomes (KDIGO) Consensus Conference. *Kidney Int* 2021;**100**:516–26. doi:10.1016/j.kint.2021.06.028

11 Gama RM, Clery A, Griffiths K, *et al.* Estimated glomerular filtration rate equations in people of self-reported black ethnicity in the United Kingdom: Inappropriate adjustment for ethnicity may lead to reduced access to care. *PloS One* 2021;**16**:e0255869. doi:10.1371/journal.pone.0255869

12 Glaser N, Deckert A, Phiri S, *et al.* Comparison of Various Equations for Estimating GFR in Malawi: How to Determine Renal Function in Resource Limited Settings? *PLOS ONE* 2015;**10**:e0130453. doi:10.1371/journal.pone.0130453

For peer review only - http://bmjopen.bmj.com/site/about/guidelines.xhtml

13 Eastwood JB, Kerry SM, Plange-Rhule J, *et al.* Assessment of GFR by four methods in adults in Ashanti, Ghana: the need for an eGFR equation for lean African populations. *Nephrol Dial Transplant* 2010;**25**:2178–87. doi:10.1093/ndt/gfp765

14 Pottel H, Delanaye P, Weekers L, *et al.* Age-dependent reference intervals for estimated and measured glomerular filtration rate. *Clin Kidney J* 2017;**10**:545–51. doi:10.1093/ckj/sfx026

15 Levey AS, Coresh J, Greene T, *et al.* Using standardized serum creatinine values in the modification of diet in renal disease study equation for estimating glomerular filtration rate. *Ann Intern Med* 2006;**145**:247–54. doi:10.7326/0003-4819-145-4-200608150-00004

16 Pattaro C. nephro: Utilities for Nephrology. 2017.https://CRAN.R-project.org/package=nephro (accessed 6 Mar 2022).

17 Diao JA, Wu GJ, Taylor HA, *et al.* Clinical Implications of Removing Race From Estimates of Kidney Function. *JAMA* 2021;**325**:184–6. doi:10.1001/jama.2020.22124

18 R: The R Project for Statistical Computing. https://www.r-project.org/ (accessed 24 May 2022).

[dataset] 19 Carey, Laura. PARIS study dataset and code. 2022.https://github.com/careyla/PARIS (accessed 21 Sep 2022).

20 Field N, Cohen T, Struelens MJ, *et al.* Strengthening the Reporting of Molecular Epidemiology for Infectious Diseases (STROME-ID): an extension of the STROBE statement. *Lancet Infect Dis* 2014;**14**:341–52. doi:10.1016/S1473-3099(13)70324-4

21 Shepherd BE, Rebeiro PF, Caribbean, Central and South America network for HIV epidemiology. Brief Report: Assessing and Interpreting the Association Between Continuous Covariates and Outcomes in Observational Studies of HIV Using Splines. *J Acquir Immune Defic Syndr 1999* 2017;**74**:e60–3. doi:10.1097/QAI.0000000000001221

22 Faul F, Erdfelder E, Buchner A, *et al.* Statistical power analyses using G*Power 3.1: Tests for correlation and regression analyses. *Behav Res Methods* 2009;**41**:1149–60. doi:10.3758/BRM.41.4.1149

23 Susantitaphong P, Cruz DN, Cerda J, *et al.* World incidence of AKI: a meta-analysis. *Clin J Am Soc Nephrol CJASN* 2013;**8**:1482–93. doi:10.2215/CJN.00710113

24 Uchino S, Kellum JA, Bellomo R, *et al.* Acute renal failure in critically ill patients: a multinational, multicenter study. *JAMA* 2005;**294**:813–8. doi:10.1001/jama.294.7.813

For peer review only - http://bmjopen.bmj.com/site/about/guidelines.xhtml

25 Schiffl H. Renal recovery from acute tubular necrosis requiring renal replacement therapy: a prospective study in critically ill patients. *Nephrol Dial Transplant Off Publ Eur Dial Transpl Assoc - Eur Ren Assoc* 2006;**21**:1248–52. doi:10.1093/ndt/gfk069

26 Schrier RW, Wang W. Acute renal failure and sepsis. *N Engl J Med* 2004;**351**:159–69. doi:10.1056/NEJMra032401

27 Fabian J, Kalyesubula R, Mkandawire J, *et al.* Measurement of kidney function in Malawi, South Africa, and Uganda: a multicentre cohort study. *Lancet Glob Health* 2022;**10**:e1159–69. doi:10.1016/S2214-109X(22)00239-X

28 Nakanga WP, Prynn JE, Banda L, *et al.* Prevalence of impaired renal function among rural and urban populations: findings of a cross-sectional study in Malawi. *Wellcome Open Res* 2019;**4**:92. doi:10.12688/wellcomeopenres.15255.1

29 Stanifer JW, Jing B, Tolan S, *et al.* The epidemiology of chronic kidney disease in sub-Saharan Africa: a systematic review and meta-analysis. *Lancet Glob Health* 2014;**2**:e174-181. doi:10.1016/S2214-109X(14)70002-6

30 Fabian J, George JA, Etheredge HR, *et al.* Methods and reporting of kidney function: a systematic review of studies from sub-Saharan Africa. *Clin Kidney J* 2019;**12**:778–87. doi:10.1093/ckj/sfz089

31 ElHafeez SA, Bolignano D, D'Arrigo G, *et al.* Prevalence and burden of chronic kidney disease among the general population and high-risk groups in Africa: a systematic review. *BMJ Open* 2018;**8**:e015069. doi:10.1136/bmjopen-2016-015069

FIGURE LEGEND

Figure 1: Kaplan-Meier estimate of survival function following hospital admission with infection.
a HR for death according to HIV status from Cox proportional hazards model adjusted for age and qSOFA score with 95% confidence intervals.
b HR for death according to AKI status from Cox proportional hazards model adjusted for age and qSOFA score with 95% confidence intervals.
HR = hazard ratio, qSOFA = quick sepsis related organ failure assessment score.

For peer review only - http://bmjopen.bmj.com/site/about/guidelines.xhtml

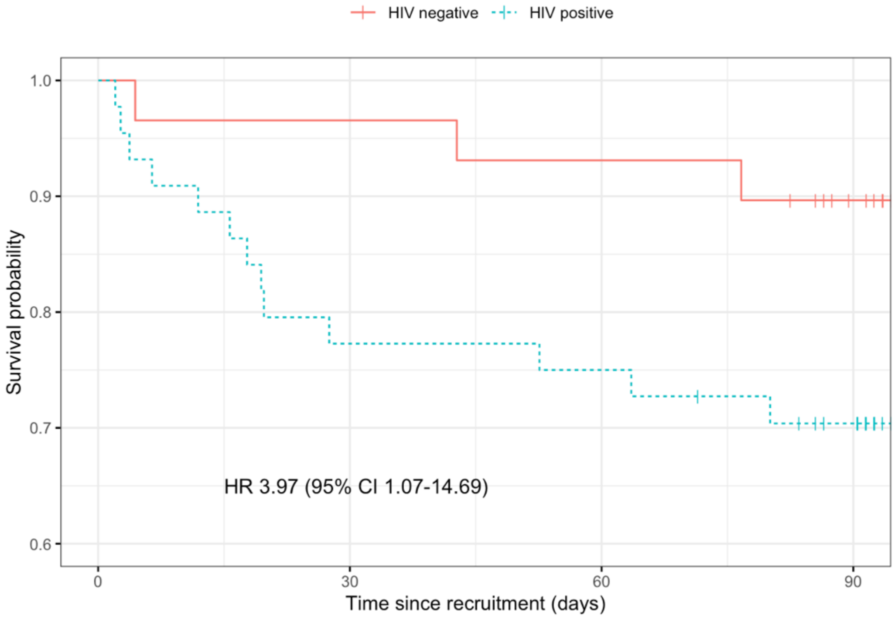

a

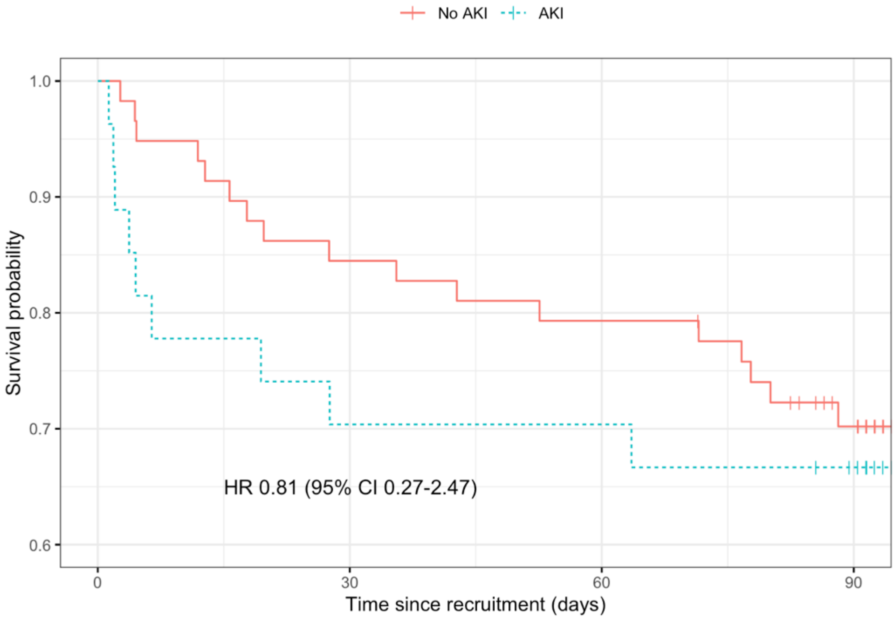

b

Figure 1: Kaplan-Meier estimate of survival function following hospital admission with infection.
a HR for death according to HIV status from Cox proportional hazards model adjusted for age and qSOFA score with 95% confidence intervals.
b HR for death according to AKI status from Cox proportional hazards model adjusted for age and qSOFA score with 95% confidence intervals.
HR = hazard ratio, qSOFA = quick sepsis related organ failure assessment score.

Title: Prospective cohort study to identify prevalence, risk factors and outcomes of infection associated kidney disease in a regional hospital in Malawi.

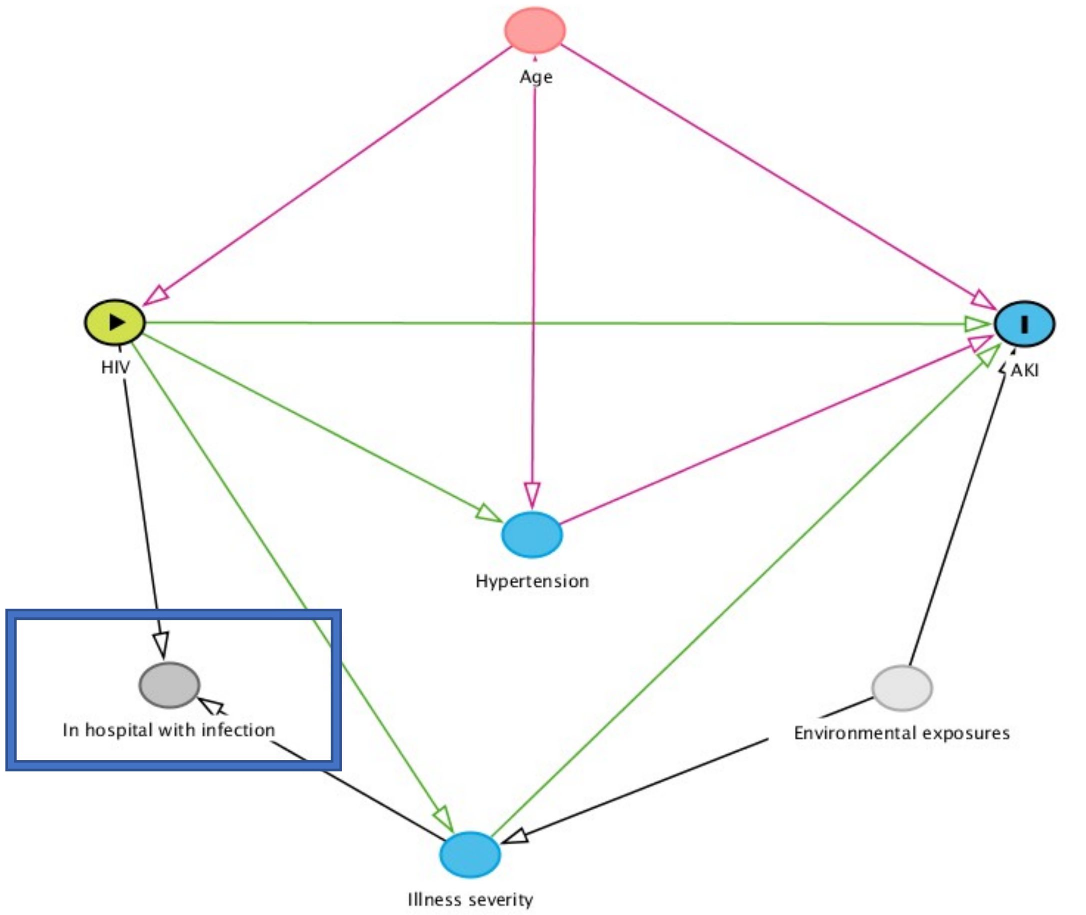

Supplementary figure 1: directed acyclic graph*.
Demonstrates the hypothesized causal relationship for examining the total effect of HIV exposure on acute kidney injury and requires adjustment for age. Note existence of collider variable *in hospital with infection*, conditioned on due to study design, by recruiting patients hospitalised with infection.
*created using DAGitty[1].

For peer review only - http://bmjopen.bmj.com/site/about/guidelines.xhtml

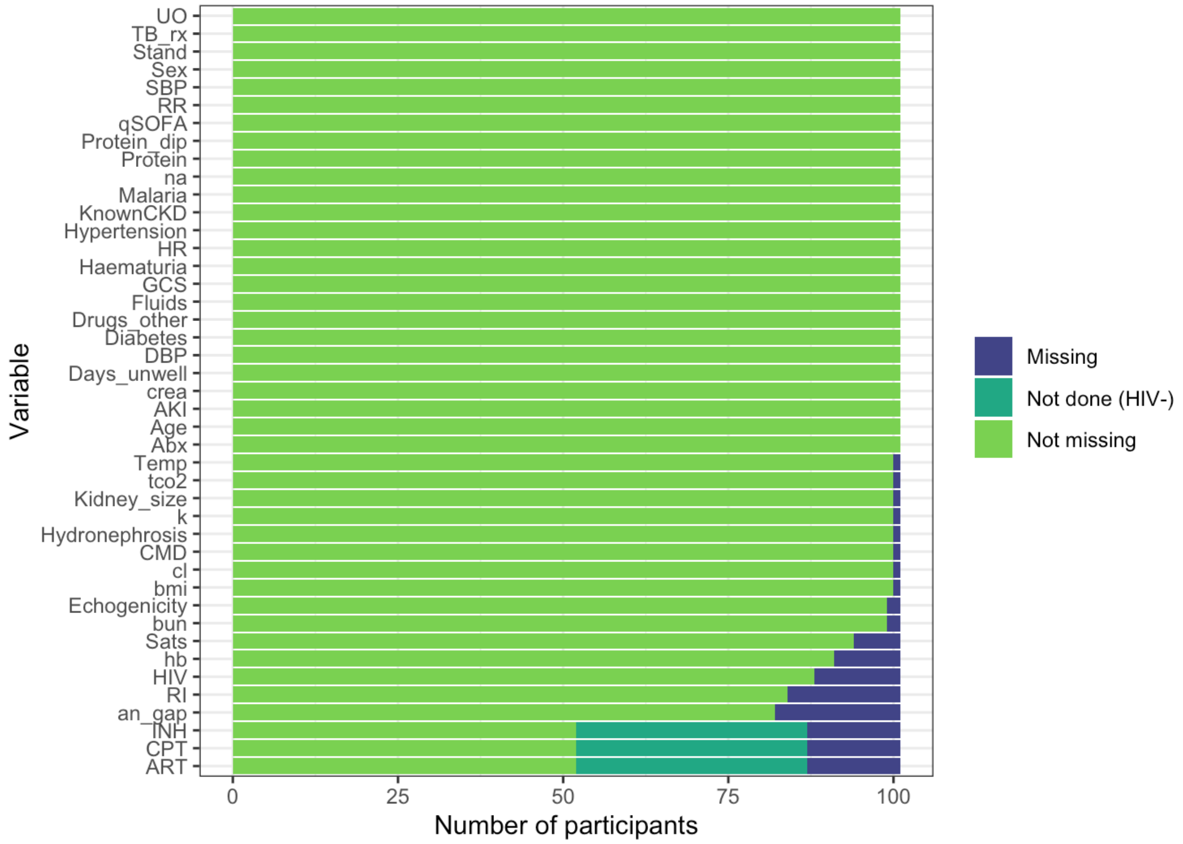

Supplementary figure 2: **missing data by variable.** HIV related variables were not available for adults without HIV, recorded as "Not done (HIV-)". Urine = able to pass urine), TB_rx = history of TB treatment, SBP = systolic blood pressure, RR = respiratory rate, HR = heart rate, drugs_other = received any over the counter or traditional medications, DBP = diastolic blood pressure, Abx = received antibiotics prior, stand = able to stand, K = serum potassium, CMD = corticomedullary differentiation, bicarb = bicarbonate, BUN = blood urea nitrogen, Sats = capillary oxygen saturations, Hb = haemoglobin, RI = renal resistive index, INH = isoniazid, CPT = cotrimoxazole preventive therapy, ART = antiretroviral therapy.

For peer review only - http://bmjopen.bmj.com/site/about/guidelines.xhtml

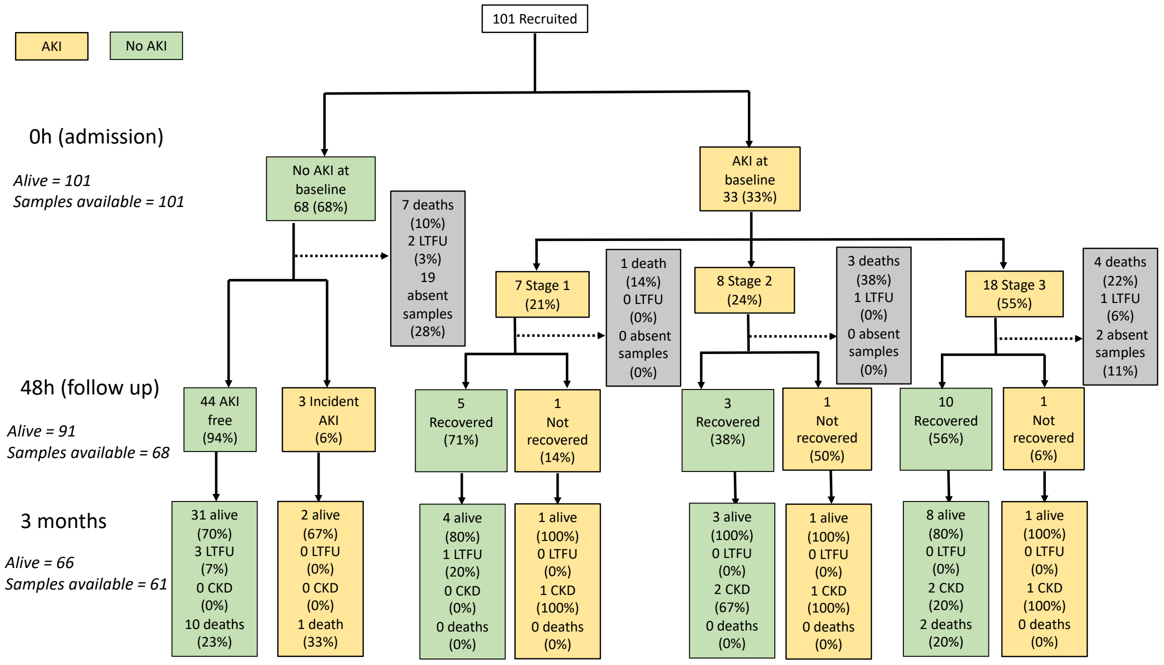

Supplementary figure 3: study flow diagram. Adults admitted to Zomba District Hospital with community acquired infection were recruited (n=101). AKI was defined using the KDIGO criteria based on a 1.5 fold increase in admission creatinine from estimated baseline, stratified by stages 1-3. AKI recovery was assessed at 48h (decrease of 0.3 mg/dL between baseline and 48 hours or a normal creatinine (< 1.3 mg/dL) at 3 months). New (incident) AKI was assessed at 48 hours and diagnosed according to KDIGO using estimated baseline creatinine. CKD was assessed at 3 months and defined as an estimated glomerular filtration rate < 60 mL/min/1.73/m^2 (composite of both CKD-EPI and MDRD equation).

LTFU = lost to follow up.

For peer review only - http://bmjopen.bmj.com/site/about/guidelines.xhtml

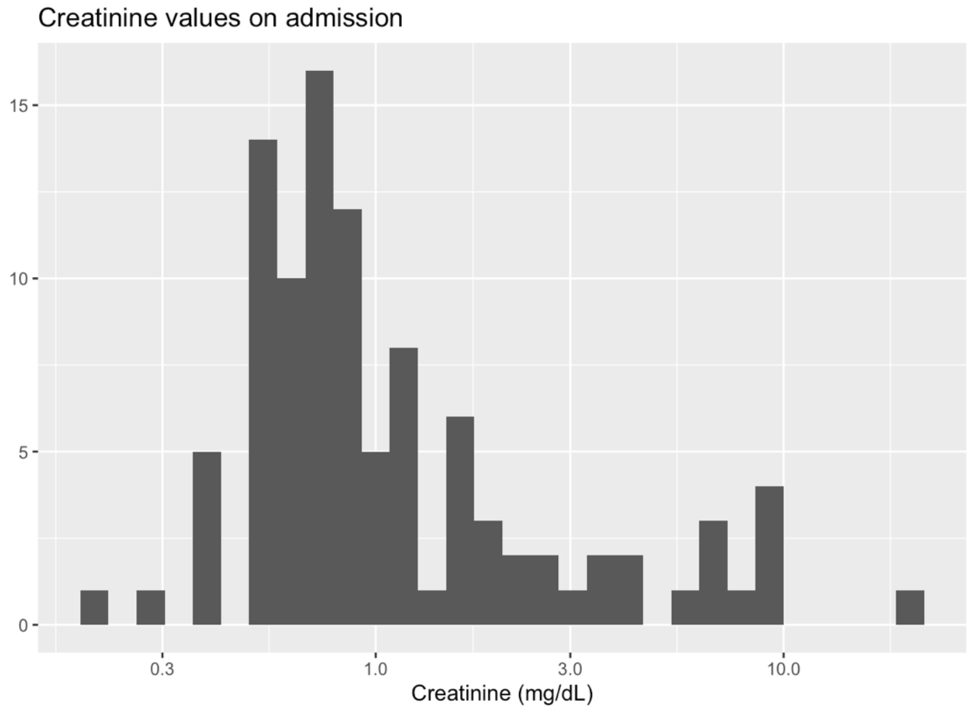

Supplementary figure 4: histogram of baseline creatinine values in mg/dL (log scale).

a

HIV -ve	qSOFA score		
**Age (years)**	**1**	**2**	**3**
**20**	0.09	0.30	1.00
**40**	0.02	0.09	0.30
**60**	0.09	0.30	1.00
**80**	0.82	2.85	10.11

b

HIV +ve	qSOFA score		
**Age (years)**	**1**	**2**	**3**
**20**	0.45	1.50	5.25
**40**	0.12	0.43	1.50
**60**	0.45	1.50	5.25
**80**	4.26	13.29	49.00

Supplementary figure 5
a AKI odds for HIV negative participants with diastolic blood pressure held constant at 80 mm Hg

For peer review only - http://bmjopen.bmj.com/site/about/guidelines.xhtml

b AKI odds for people living with HIV with diastolic blood pressure held constant at 80 mm Hg. Colour coding yellow to orange depicts increasing odds.

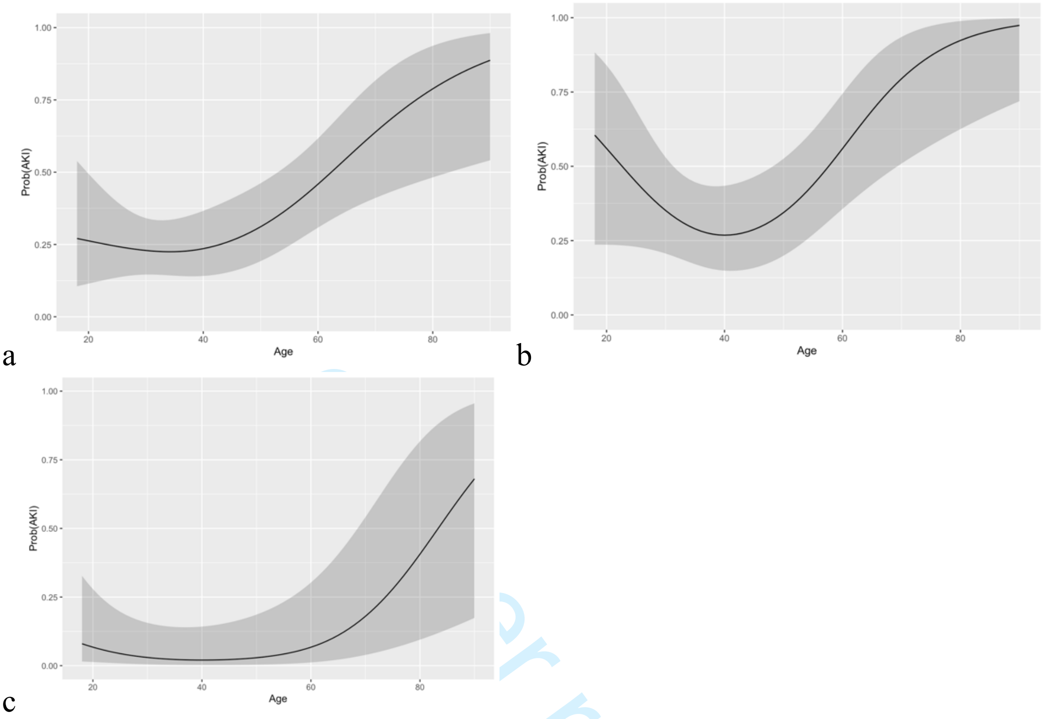

Supplementary figure 6
a unadjusted effect of age on probability of AKI. Age is expressed as a spline term with 3 knots.
b adjusted effect of age on probability of AKI. Diastolic blood pressure = 75 mm Hg, qSOFA score = 2, HIV status = positive.
c Adjusted effect of age on probability of AKI. Diastolic blood pressure = 75 mm Hg, qSOFA score = 1, HIV status = negative.

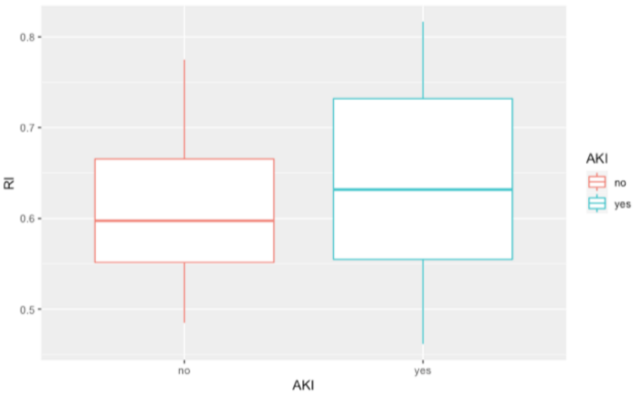

Supplementary figure 7: boxplot of RI distributions by AKI status
RI = renal resistive index.

Supplementary table 1: ultrasound, laboratory and derived indices.

Variable		Value
Ultrasound	Kidney Size (cm)	10 [9-10]
	Hydronephrosis* n/N (%)	16/100 (16%)
	Loss of corticomedullary differentiation* n/N (%)	44/100 (44%)
	Increased echogenicity† n/N (%)	41/99 (41%)
	Renal Resistive Index	0.62 [0.55-0.68]
Urine	Able to pass urine n/N (%)	97/101 (96%)
	Proteinuria on dipstick n/N (%)	29/101 (29%)
	Proteinuria (mg/dL)	0.0 [0.0-138.5]
	Microscopic Haematuria n/N (%)	54/101 (53%)
Observations	Systolic blood pressure (mm Hg)	117 [103-133]
	Diastolic blood pressure (mm Hg)	75 [64-84]
	Heart rate (beats/min)	101 [84-123]
	Temperature (°C)	37 [37-38]
	Oxygen saturation (%)	98 [96-99]
	Respiratory rate (breaths/min)	24 [22-28]
	Glasgow coma score < 15 n/N (%)	27/101 (27%)
Illness severity	Unable to stand unaided n/N (%)	27/101 (27%)
	Length of time unwell for (days)	7.0 [3.0-21.0]
	Receiving intravenous fluid n/N (%)	63/101 (62%)
	qSOFA score	2.0 [2.0-2.0]
Laboratory parameters	Haemoglobin (mmol/L)	11.9 [8.8-13.9]
	Blood Urea Nitrogen (mmol/L)	15 [8-30]
	Chloride (mmol/L)	104 [99-108]
	Sodium (mmol/L)	136 [131-140]
	Potassium (mmol/L)	3.6 [3.3-4.5]
	Bicarbonate (mmol/L)	23 [19-26]
	Creatinine (mmol/L)	0.80 [0.60-1.60]
	Anion Gap (mmol/L)	16 [15-18]

Values are median [interquartile range].
* Hydronephrosis/corticomedullary differentiation missing for 1 participant n=100
† Echogenicity missing n=2

For peer review only

Supplementary table 2: demographic univariable associations with AKI status.

Variable	No AKI	AKI	*p* value
Age† (years)	35.75 [28.62-46.06]	46.46 [35.44-65.43]	0.01
Male sex* n (%)	32 (47)	15 (45)	1.00
Body mass index (kg m^{-2})	22.06 [19.38-23.63]	20.96 [18.87-23.23]	0.19
Creatinine mg/dL	0.7 (0.5-0.8)	2.7 (1.6-6.3)	<0.01**
HIV infected* n (%)	33 (55)	20 (71)	0.29
Receiving antiretroviral therapy* n (%)	26 (79)	16 (84)	0.39
Receiving co-trimoxazole preventative therapy* n (%)	25 (76)	15 (79)	0.52
Receiving isoniazid preventative therapy* n (%)	6 (18)	5 (26)	0.33
Received TB treatment* n (%)	6 (9)	5 (15)	0.33
Prior Malaria* n (%)	12 (18)	9 (27)	0.30
Diagnosis of Diabetes* n (%)	0 (0)	1 (3)	0.33
Diagnosis of Hypertension* n (%)	2 (3)	5 (15)	0.04
Antibiotic use prior* n (%)	51 (75)	29 (88)	0.19
Over the counter/traditional meds* n (%)	2 (3)	2 (6)	0.60
Vomiting* n (%)	14 (21)	11 (33)	0.22
Diarrhoea* n (%)	10 (15)	6 (18)	0.77
Cough* n (%)	26 (38)	17 (52)	0.28

Values are median [interquartile range].
* Fisher's exact test † Wilcoxon rank sum

For peer review only

Supplementary table 3: ultrasound and urine related univariable associations with AKI status.

**Variable**	**No AKI**	**AKI**	***p* value**
Kidney Size† (cm), median (IQR)	9.8 (8.96-10.49)	9.63 (8.67-10.35)	0.65
Hydronephrosis*, n (%)	9 (13%)	7 (21%)	0.39
Loss of corticomedullary differentiation*, n (%)	28 (42%)	16 (48%)	0.53
Increased echogenicity*, n (%)	25 (38%)	16 (48%)	0.29
Renal Resistive Index†, median (IQR)	0.6 (0.55-0.66)	0.63 (0.56-0.73)	0.19
Able to pass urine*, n (%)	67 (99%)	30 (91%)	0.10
Proteinuria on dipstick*, n (%)	11 (16%)	18 (55%)	<0.01
Proteinuria† (mg/dL), median (IQR)	0 (0-26.4)	182.1 (65.4-241.7)	<0.01
Microscopic Haematuria*, n (%)	27 (40%)	27 (82%)	<0.01

Values are median [interquartile range].
* Fisher's exact test † Wilcoxon rank sum

For peer review only - http://bmjopen.bmj.com/site/about/guidelines.xhtml

Supplementary table 4: observation and laboratory derived univariable associations according to AKI status.

Variable	No AKI	AKI	*p* value
Temperature† (°C)	37.2 [36.85-37.8]	37 [36.8-37.8]	0.58
Heart rate† (beats/min)	99.5 [83.75-124]	102 [92-123]	0.51
Respiratory rate† (breaths/min)	23.5 [20-26.25]	26 [23-29]	0.08
Systolic blood pressure† (mm Hg)	115.5 [103.75-128.25]	118 [101-141]	0.28
Diastolic blood pressure† (mm Hg)	74 [64-83.25]	77 [63-87]	0.35
Oxygen saturation† (%)	98 [96-99]	97 [95.5-98]	0.07
Glasgow coma score < 15*, n (%)	17 (25)	10 (30)	0.63
Unable to stand unaided*, n (%)	15 (22)	12 (36)	0.15
Length of time unwell† (days)	9 [3-30.44]	5 [3-14]	0.18
Receiving intravenous fluid*, n (%)	38 (56)	25 (76)	0.08
qSOFA score†	2 [2-2]	2 [2-2]	0.27
Haemoglobin† (mmol/L)	11.9 [9.2-13.75]	11.55 [8.1-14.6]	0.88
Blood Urea Nitrogen† (mmol/L)	10 [7-17.5]	40.5 [22.75-82.25]	<0.01
Chloride† (mmol/L)	102.5 [98-108]	105.5 [101.75-109.75]	0.09
Sodium† (mmol/L)	136 [130.75-140]	136 [131-140]	0.99
Potassium† (mmol/L)	3.6 [3.3-4.2]	3.6 [3.17-5.18]	0.80
Bicarbonate† (mmol/L)	24 [22-26]	16 [14-23]	<0.01
Creatinine† (mg/dL)	0.7 [0.5-0.8]	2.7 [1.6-6.3]	<0.01
Anion Gap† (mmol/L)	16.5 [15-18]	16.5 [15-20]	0.24
eGFR† (ml/min/1.73/m^2) (MDRD)	124.04 [92.68-159.04]	22.95 [11.21-40.24]	<0.01
Creatinine clearance† (mL/min) (Cockroft and Gault)	118.25 [93.71-143.71]	24.88 [14.48-33.66]	<0.01
Chronic kidney disease at 3 months*, n (%)	5 (12)	7 (37)	0.06

Values are median [interquartile range].
* Fisher's exact test † Wilcoxon rank sum
MDRD = 4-parameter Modification of Diet in Renal Disease equation

For peer review only

Supplementary Table 5: Effect of age on AKI when full age spectrum equation is used to back calculate creatinine, using assumed eGFR 100.

Odds ratios and 95% confidence intervals for AKI		
Age in years	Unadjusted	Adjusted for age and HIV status
20	1.17 (0.38-5.63)	2.23 (1.28-38.11)
30	0.91 (0.57-1.68)	0.91 (0.71-2.34)
40 (ref)	1.00	1.00
50	1.92 (1.45-3.07)	2.03 (1.49-4.43)
60	5.43 (3.22-20.11)	8.72 (4.21-171.31)
70	17.46 (7.20-189.53)	64.33 (18.65-28395.00)
80	56.58 (15.97-2103.49)	529.11 (96.75-5708002.00)

Supplementary Table 6: Effect of age on AKI and AKD according to each definition on mortality*.

	MDRD back calculation assumed eGFR 100 mL/min		*p* value
	No AKI	AKI	
Death by 3 months, n (%)	18/68 (26%)	10/33 (30%)	0.70
	**Full age spectrum equation assumed eGFR 100 mL/min**		***p* value**
	**No AKI**	**AKI**	
Death by 3 months, n (%)	17/65 (26%)	11/36 (30%)	0.60
	**Lowest creatinine as baseline**		***p* value**
	**No AKI**	**AKI**	
Death by 3 months, n (%)	28/84 (33%)	0/17 (0%)	<0.01
	**Acute kidney disease**		***p* value**
	**No AKD**	**AKD**	
Death by 3 months, n (%)	11/38 (29%)	17/63 (27%)	0.70

**p* values obtained by Chi squared test of significance.
AKD = acute kidney disease, AKI = acute kidney injury

For peer review only - http://bmjopen.bmj.com/site/about/guidelines.xhtml

Reference

1. Textor J, van der Zander B, Gilthorpe MS, Liśkiewicz M, Ellison GTH. Robust causal inference using directed acyclic graphs: the R package 'dagitty.' *Int J Epidemiol*. Published online January 15, 2017:dyw341. doi:10.1093/ije/dyw341

For peer review only

For peer review only - http://bmjopen.bmj.com/site/about/guidelines.xhtml

STROBE cohort study checklist:

Prospective cohort study to identify prevalence, risk factors and outcomes of infection associated kidney disease in a regional hospital in Malawi.

Description	Item number	Recommendation	Present?	Where described
Title and abstract	1	(*a*) Indicate the study's design with a commonly used term in the title or the abstract	Yes	Abstract p2
		(*b*) Provide in the abstract an informative and balanced summary of what was done and what was found	Yes	Abstract p2
Introduction				
Background/rationale	2	Explain the scientific background and rationale for the investigation being reported	Yes	Introduction p4
Objectives	3	State specific objectives, including any prespecified hypotheses	Yes	Introduction p5
Methods				
Study design	4	Present key elements of study design early in the paper	Yes	Methods p5-6
Setting	5	Describe the setting, locations, and relevant dates, including periods of recruitment, exposure, follow-up, and data collection	Yes	Abstract p2<br>Methods p5-10<br>Results p11
Participants	6	(*a*) Give the eligibility criteria, and the sources and methods of selection of participants. Describe methods of follow-up	Yes	Methods p6-7
		(*b*) For matched studies, give matching criteria and number of exposed and unexposed	NA	NA
Variables	7	Clearly define all outcomes, exposures, predictors, potential confounders, and effect modifiers. Give diagnostic criteria, if applicable	Yes	Methods p7-9<br>Supplement p1
Data sources/ measurement	8	For each variable of interest, give sources of data and details of methods of assessment (measurement). Describe comparability of assessment methods if there is more than one group	Yes	Methods p6-10
Bias	9	Describe any efforts to address potential sources of bias	Yes	Methods p8-10<br>Supplement p1
Study size	10	Explain how the study size was arrived at	Yes	Methods p10

For peer review only

Quantitative variables	11	Explain how quantitative variables were handled in the analyses. If applicable, describe which groupings were chosen and why	Yes	Methods p9
Results				
Participants	13	(a) Report numbers of individuals at each stage of study—eg numbers potentially eligible, examined for eligibility, confirmed eligible, included in the study, completing follow-up, and analysed	Yes	Supplementary figure 3, p3
		(b) Give reasons for non-participation at each stage	Yes	Methods p10, Supplementary figure 3, p3
		(c) Consider use of a flow diagram	Yes	Supplementary figure 3, p3
Descriptive data	14	(a) Give characteristics of study participants (eg demographic, clinical, social) and information on exposures and potential confounders	Yes	Results p11
		(b) Indicate number of participants with missing data for each variable of interest	Yes	Supplement p2
		(c) Summarise follow-up time (eg, average and total amount)	Yes	Results p11
Outcome data	15	Report numbers of outcome events or summary measures over time	Yes	Results p13
Main results	16	(*a*) Give unadjusted estimates and, if applicable, confounder-adjusted estimates and their precision (eg, 95% confidence interval). Make clear which confounders were adjusted for and why they were included	Yes	Results p15 Supplement p1
		(*b*) Report category boundaries when continuous variables were categorized	NA	NA
		(*c*) If relevant, consider translating estimates of relative risk into absolute risk for a meaningful time period	NA	NA
Other analyses	17	Report other analyses done—eg analyses of subgroups and interactions, and sensitivity analyses	NA	Supplement p10
Discussion				
Key results	18	Summarise key results with reference to study objectives	Yes	Discussion p16-17

For peer review only

Limitations	19	Discuss limitations of the study, taking into account sources of potential bias or imprecision. Discuss both direction and magnitude of any potential bias	Yes	Discussion p19-20
Interpretation	20	Give a cautious overall interpretation of results considering objectives, limitations, multiplicity of analyses, results from similar studies, and other relevant evidence	Yes	Discussion p20
Generalisability	21	Discuss the generalisability (external validity) of the study results	Yes	Discussion p17-18
Other information				
Funding	22	Give the source of funding and the role of the funders for the present study and, if applicable, for the original study on which the present article is based	Yes	Funding p21

For peer review only
